# Rosemary essential oil nanoparticles enhance antimicrobial activity and induce bacterial DNA disruption

**DOI:** 10.1007/s10123-026-00815-2

**Published:** 2026-05-13

**Authors:** Habiba A. Ahmed, Hala El-Adawi, Asmaa Abdel-Fattah, Abeer E. Abd El-Wahab

**Affiliations:** 1 Plant Biochemistry Department, National Research Centre, Dokki, 12622 Giza Egypt; 2 Medical Biotechnology Department, Genetic Engineering and Biotechnology Institute, City of Scientific Research and Technological Applications (SRTA-City), New Borg EL-Arab, Alexandria, 21934 Egypt; 3 Pharmaceutical and Fermentation Industries Development Center, City of Scientific Research and Technological Applications (SRTA-City), New Borg EL-Arab, Alexandria, 21934 Egypt

**Keywords:** Nano-formulation, Gum Arabic, Silver nanoparticles, Multidrug-resistant bacteria, Antibacterial activity, Cytocompatibility, DNA damage

## Abstract

The current work aimed to formulate Rosemary essential oil nanoparticles (Ros-ES-NPs) and evaluate their antimicrobial effect in comparison with free oil (Ros- ES). Physicochemical characterization was performed using Fourier-transform infrared spectroscopy (FTIR), dynamic light scattering, zeta potential analysis, transmission electron microscop y (TEM), scanning electron microscopy (SEM), and thermogravimetric analysis (TGA). The cytotoxic activity of Ros-ES-NPs on human lung fibroblast cell line (Wi-38) was examined in comparison with Ros- ES using MTT assay. Ros-ES exhibited dose-dependent cytotoxicity, with an IC_50_ of approximately 6.06 mg/ml on Wi-38. In contrast, the Ros-ES-NPs not only showed no toxicity but actually appeared to increase cell viability (up to ≈ 136%) possibly due to high metabolic actvity.

The antimicrobial activity of Ros-ES and Ros-ES-NPs was evaluated against three isolates of Multidrug-Resistant Bacteria, comprising two Gram-negative isolates (*Shigella flexneri and Klebsiella pneumoniae*) and one Gram-positive isolate (*Streptococcus pneumoniae*), alongside C*andida albicans* as the fungal pathogen. Ros-ES-NPs showed a significant antimicrobial efficacy against all isolates under study where, at 25 mg/ml, Ros-ES inhibited *K. pneumoniae and Shigella flexneri* by 55.16 ± 0.09% and 76.47 ± 1.16%, respectively, whereas Ros-ES-NPs showed inhibition rates of 95.66 ± 9.74% and 87.13 ± 2.63%, respectively. Against *Streptococcus pneumoniae*, at 25 mg/ml, Ros-ES inhibited by 68.75 ± 0.60%, while Ros-ES-NPs exhibited a higher inhibition rate of 80.73 ± 5.6%. In case of *C. albicans*, Ros-Es showed a higher inhibition (81.85 ± 0.54) than Ros-Es-NPs (60.39 ± 5.69). Rosemary essential oil nano-formulation significantly enhances antibacterial efficacy while improving biocompatibility and inducing pronounced microbial DNA damage.

**Supplementary Information:**

The online version contains supplementary material available at https://doi.org/10.1007/s10123-026-00815-2.

## Introduction

One of the biggest risks to human health in the twenty-first century is antimicrobial resistance (AMR), which has emerged as a major problem in the battle against harmful microbes. A study published in 2022 found that global AMR-related deaths in 2019 were higher than those caused by HIV/AIDS or malaria, directly leading to 1.2 million deaths and playing a role in a further 4.95 million deaths (Murray et al. 2022). According to reports, almost 80% of people worldwide already use plant-based or herbal medicines (Swamy et al. 2016). Over 9000 plant species have been found for their medicinal properties, while about 1500 species have been identified for their aromatic properties (Margaris et al. 1982).

Essential oils (EOs) are naturally occurring components of aromatic and therapeutic plants that are rich in bioactive substances and metabolites capable of effectively treating a wide range of diseases, including microbial infections. All plant parts, including flowers, leaves, roots, bark, and seeds, may contain them (Bakkali et al. 2008). Rosemary (*Rosmarinus officinalis L*.) essential oil is a complex mixture of volatile bioactive compounds, primarily monoterpenes such as 1,8-cineole, α-pinene, camphor, and borneol, along with sesquiterpenes and phenolic compounds like rosmarinic acid (Ojeda-Sana et al. 2013). These constituents are largely responsible for its antibacterial, antifungal, antioxidant, and flavoring properties (Stojiljkovic et al. 2018). However, many of these compounds are thermally unstable and prone to oxidation or volatilization, which can reduce the efficacy of the oil (Presti et al. 2005).

Formulating rosemary EO as a nano-emulsion or nanoparticle-based system can enhance its stability, bioavailability, and antimicrobial potency (Campolo et al. 2020), making it more suitable for therapeutic and industrial applications. Essential oils can be extracted from aromatic plants using steam distillation, hydrodistillation, dry distillation, or mechanical methods (Reyes-Jurado et al. 2015). However, their high hydrophobicity, low solubility in water, and volatility limit their applications (Wińska et al. 2019).

Over the past 20 years, nanotechnology-based approaches have been developed to enhance the bioavailability and dispersity of plant EOs in aqueous systems (Anarjan et al. 2014, 2017; Jafari and McClements 2018). Reducing particle size to the nanoscale increases surface area, solubility, and dissolution rate (Anarjan et al. 2017). Various nanoscale formulations, including nanoparticles, nanocapsules, nanodroplets, nanoemulsions, and nanoliposomes, have been prepared (Jafari and McClements 2018).

In oil-in-water (O/W) nanoemulsions, EO droplets are dispersed in water with emulsifiers such as lecithin, gum Arabic, sodium caseinate, xanthan gum, Tween 20, Tween 80, and saponin (Jafari and McClements 2018; Xue et al. 2015; Qiu et al. 2015; Vijaya et al. 2015; Niu et al. 2016; Chong et al. 2018; Ryu et al. 2010). O/W nanoemulsions are widely used in cosmetics, pharmaceuticals, personal care, agrochemicals, and food supplements, with droplet sizes ranging from 100 to 1000 nm (Anarjan et al. 2017; Jafari and McClements 2018). These emulsions can be prepared using high-energy methods (ultrasonic emulsification, high-pressure homogenization, high-shear stirring, and membrane emulsification) or low-energy techniques (spontaneous emulsification, emulsion inversion point, phase inversion composition/temperature) (Chong et al. 2018; Ryu et al. 2010; Sayyar and Jafarizadeh-Malmiri 2019).

Unlike previously reported rosemary essential oil nano-formulations that primarily focus on improved dispersion and membrane-level antimicrobial effects, the present study demonstrates that a gum Arabic–stabilized nano-architecture incorporating trace silver nanoparticles fundamentally alters the biological interaction of rosemary oil with microorganisms, leading to reproducible damage of microbial DNA, while maintaining mammalian cell compatibility. This mechanistic shift highlights a new functional dimension for plant-based nano-antimicrobials.” By combining physical characterization (FTIR, TEM, SEM, zeta potential, TGA) with biological evaluation, this work provides a comprehensive understanding of the structure–function relationship of the nanoparticles and offers a promising strategy for safe and effective nano-based antimicrobial therapies.

## Materials and methods

All chemicals used in this study were purchased from Sigma (USA) and Fluka (Switzerland) and were of analytical grade. Dried leaves of rosemary were obtained from the local market in Alexandria, Egypt. For DNA isolation, Genomic DNA Purification Kit was purchased from Promega. Human lung fibroblast cell line (Wi-38) was obtained from our Institute (GEBRI). Rosemary essential oil was extracted using a water distillation technique.

### Multi drug microbial isolates

*Streptococcus pneumoniae*, *Shigella flexneri*,* Klebsiella pneumoniae*,* and Candida albicans* were clinical isolates, acquired from Mibaret Al-Asafra hospital -Alexandria, Egypt. Bacterial isolates were with high rates of multi-drug resistance, including resistance to ampicillin, tetracycline, and trimethoprim-sulfamethoxazole. *Candida albicans* was non-susceptible to at least one antifungal drug in two different drug classes.

### Antimicrobial activity

#### Microplate reader assay

The antibacterial activity of the test samples was evaluated using a microplate reader assay technique against Gram-positive bacteria (*Streptococcus pneumoniae*), Gram-negative bacteria (*Shigella flexneri. and Klebsiella pneumoniae*), and fungi (*Candida albicans*). This assay determined both the percentage of microbial inhibition and the IC 50, following a modified protocol of (Bechert et al. 2000; Karthik et al. 2014). In brief, 100 µL of pre-inoculated pathogens (6 × 10^4^ CFU/mL) in LB broth was added to each well of a 96-well plate, followed by 100 µL of the test samples, including Ros-EO and Ros-EO-NPs, in triplicate. Plates were incubated under microaerophilic conditions at 37 °C for 24 hours. After incubation, absorbance was measured at 600 nm using an automated ELISA microplate reader. The percentage of inhibition was then calculated using the standard formula:

$$\mathrm{Inhibition}\;{\%}=\left[\left(\mathrm{A0}-\mathrm{A}\right)/\mathrm{A0}\right]\times{100}$$

Where A0 is the control absorbance, A is the treated group absorbance, and A1 (blank) is usually subtracted from both A0 and A before calculation.

##### Cytotoxicity assay

For the determination of treatments concentrations that are non-toxic to human lung fibroblast cell line (Wi-38), the cytotoxic assay was performed according to Schnitzler et al. 2001. Briefly, a cell suspension of 6 × 10^4^ cell mL‾^1^ was collected and seeded in 96-well plates (100 µL cell suspension per well). The plates were incubated at 37 °C in humidified 5% CO_2_ for 24 h. After obtaining a semi confluent cell layer, the exhausted media was discarded and 100 µL of different treatment concentrations prepared in DMEM were added. Wells containing only media were used as a negative control. The cell plates were incubated at the same growth conditions for 3 days. After 3 days, 50 µL of MTT was added to each well and incubated for 3 h at 37 °C in humidified 5% CO_2_. Viable cells reduced the yellow MTT to purple formazan crystals, which were dissolved in 200 µL DMSO. Absorbance at ~ 570 nm was measured, with the intensity of the purple color correlating to the number of viable cells. Measurements were performed in triplicates, and the concentration required for a 50% inhibition of viability (IC50) was determined graphically. The absorbance at 570 nm was measured with a microplate reader (Bio-Rad, Richmond, CA), using wells without sample containing cells as blanks.

$${\%}\;\text{cell viability}=\text{A570 of treated cells } /\text{A570 of control cells}\;\times{100}{\%}$$

##### Microbial genomic DNA preparation

The microbial genomic DNAs were prepared according to the manufacturer’s instructions (Promega). The DNA concentrations were determined by measuring the optical density (OD) at 260 and 280 nm, using an ultraviolet (UV) spectrophotometer (Ultraspec 2000, Pharmacia Biotech., UK).

##### DNA fragmentation assay

A distinctive feature of apoptosis at the biochemical level is DNA fragmentation. This method was used as a semi-quantitative method for measuring apoptosis. Such phenomenon, described for the first time by (Wyllie 1980), can be visualized by an agarose gel electrophoresis analysis. The present protocol provides a method for qualitative determination of DNA fragmentation in the presence of treatments (Ros-ES & Ros-ES-NPs).

### Extraction of rosemary essential oil

Dried leaves of rosemary (*Rosmarinus officinalis L.*) were used to extract essential oil (Ros-EO) via a water distillation technique. The powdered leaves were placed in a distillation apparatus with water and boiled, allowing the volatile compounds to evaporate, condense, and be collected as the essential oil. The obtained Ros-EO was stored in a dark glass container at − 20 °C until further use.

### Nano essential oil preparation

**Fig. 1:**
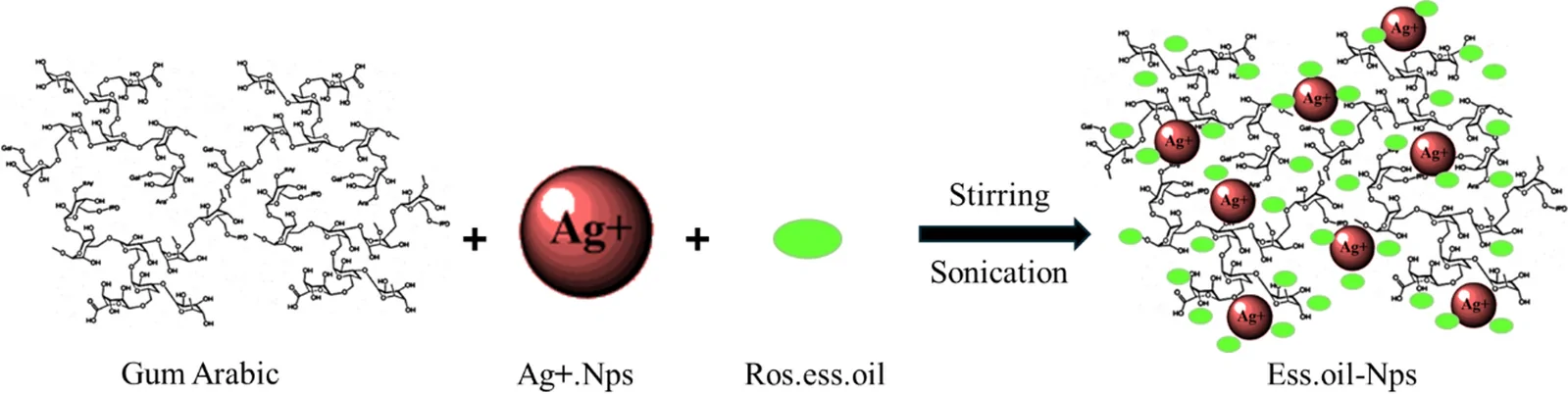
Schematic representation of the preparation of rosemary essential oil nanoparticles (Ros-Es-NPs) using gum Arabic as a polymer and silver nanoparticles (Ag-NPs)

To synthesize Arabic gum-stabilized essential oil nanoparticles (NPs) of rosemary (Ros-Es-NPs) using the freeze-drying technique (Fig. 1), ten grams of Arabic gum were dissolved in 50 ml of distilled water with stirring for 3 h. In the second step, 2.5 ml of rosemary essential oil (Ros-Es) was mixed with 2.5 ml of Tween 80, stirring for 15 min in an Amber glass bottle. The third step involved adding the previous mixture to the gum Arabic solution dropwise, while stirring. Then, 100 µL of silver nanoparticles (1,000 ppm) were added dropwise. The final mixture was sonicated for 10 min using the DAIGGER ULTRA-SONIC Model GEX 750, USA. Finally, the product was freeze-dried at -55 °C for 24 h and stored at-80 °C in an airtight container until use (Abdelaziz et al. 2024).

### Physical characterization

*Fourier Transform Infrared Spectroscopy (FTIR)*: FTIR spectra were used to detect functional groups and evaluate the degree of association between substances during nanoparticle production, with a resolution of 4.0 cm⁻¹ over the range of 4000 to 400 cm⁻¹ (Bruker Optics Tensor 27, Bruker Corporation, Billerica, MA, USA). *Transmission Electron Microscopy (TEM)*: This method was employed to investigate the morphology and particle size of the synthesized essential oil nanocomposite. The nanoparticle solution was sonicated for 5 min to improve dispersion before sample preparation. *Scanning Electron Microscopy (SEM)*: SEM was used to examine the microstructure of the essential oil nanocomposite. The lyophilized essential oil nanoparticles sample was affixed to an adhesive carbon stub, and imaging was conducted using a tabletop SEM (JEOL 6340, Japan) operating at 15 kV. *Thermogravimetric Analysis (TGA)*: The control and essential oil nanoparticles were scanned from 0 to 1000 °C at a heating rate of 10 °C/min under a flow of nitrogen using the Themsys One Plus (SETARAM, France). *Zeta Sizer*: The particle size, polydispersity index (PDI), and surface charges of the nanoparticles in water suspension at room temperature were estimated using a Malvern Zeta Sizer (Nano ZS, Malvern, UK) as described by Elbehery et al.2025 & Ahmed et al. 2025).

### Statistical analysis

Results are presented as mean ± standard deviation (SD) based on three independent experiments, each conducted in triplicate. Statistical analysis was carried out using Co Stat software for Windows (version 6.45). Differences among groups were regarded as statistically significant at *p* < 0.05.

## Results and discussion

### Fourier-transform infrared spectroscopy (FTIR)

**Fig. 2:**
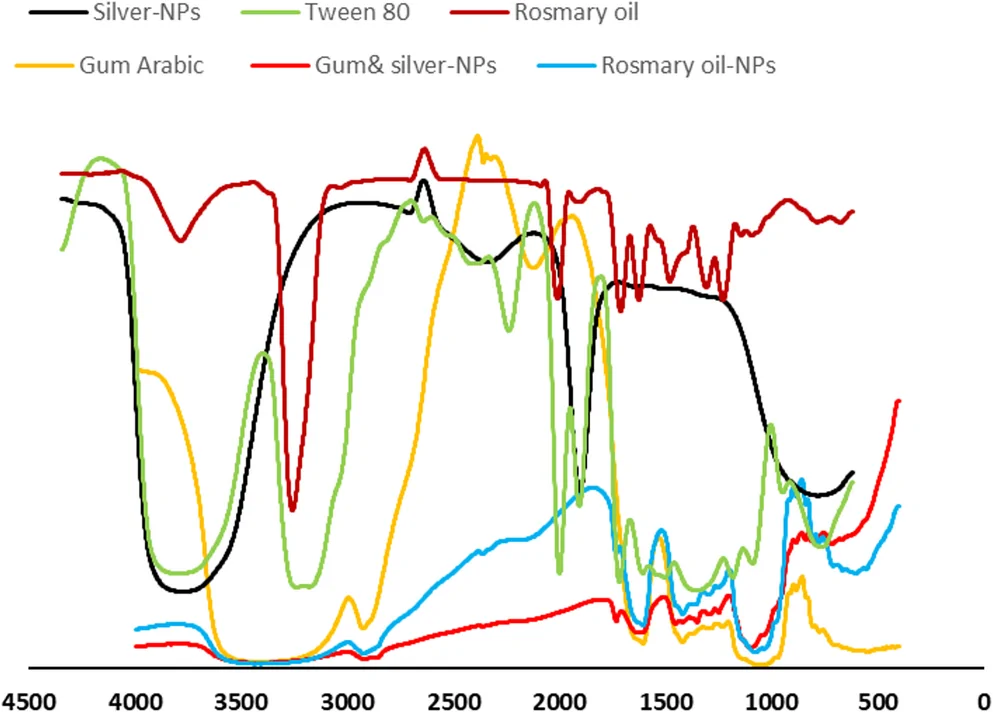
Fourier-transform infrared (FTIR) spectra of gum Arabic (orange), silver nanoparticles (black), blank formulation (gum Arabic + Ag-NPs, red), rosemary essential oil nanoparticles (Ros-Es-NPs, dark red), and native rosemary essential oil nanoparticles (blue)

FTIR is a technique used in nanoparticle studies, where the resulting spectrum reflects the level of interaction between the compounds and the nanomaterial. The FTIR spectra of the native essential oil (Ros-Es), the Nano form (Ros-Es-NPs), and all ingredients are presented in Fig. 2. The orange spectrum illustrated gum Arabic as a polymer, showing distinct peaks at 3403 cm⁻¹ corresponding to the -OH group, 2926 cm⁻¹ corresponding to -CH₂, 1605 cm⁻¹ corresponding to -C = O, 1419 cm⁻¹ corresponding to -CH, 1068 cm⁻¹ assigned to C–O–C, and 550 cm⁻¹ indicating -C–H linkages (Ahmed et al. 2023 & Lin et al. 2022). In the FTIR spectrum of Tween 80 (green spectrum), a broad and strong band was observed at 3471 cm⁻¹, attributed to the stretching vibrations of O–H. The bands at 1106 cm⁻¹ and 1733 cm⁻¹ are respectively ascribed to the asymmetric C–O and C = O stretching vibrations (Espinosa et al. 2010). In the FTIR spectrum of the purified Ag nanoparticles (black spectrum), several bands were identified: O–H stretching at approximately 3444 cm⁻¹, aldehydic C–H stretching at 2418 cm⁻¹, a C = O group at 1638 cm⁻¹, C–C and C–N stretching at 1388 cm⁻¹, and O–H stretching at 1252 cm⁻¹ (Yue et al. 2012, Ahmed et al. 2021 & Karthik et al. 2014). In the FTIR spectrum of Ros-Es (dark red spectrum), key bands were identified at 1371, 1230, 1062, 987, and 858 cm⁻¹. These bands indicate that 1, 8-cineole is the main component of Ros-Es and are attributed to the vibrations of the CH₃ (CO), C–O–C, and CH₂ groups (Ahmed et al. 2021). The FTIR spectrum of (blank) gum Arabic with silver nanoparticles (red spectrum) illustrated the overlap between the peaks of Ag nanoparticles and the gum Arabic spectrum. This overlap, compared to the separate spectra of Ag nanoparticles and gum Arabic, provides evidence of an interaction between the Ag nanoparticles and gum Arabic. The FTIR spectrum of the Ros-Es-NPs (blue spectrum) displayed overlapping peaks among Ag nanoparticles, Tween 80, gum Arabic, and essential oil, with the presence of new peaks suggesting a potential chemical reaction among all the components (Goda et al. 2025).

### Transmission Electron Microscopy (TEM)

**Fig. 3:**
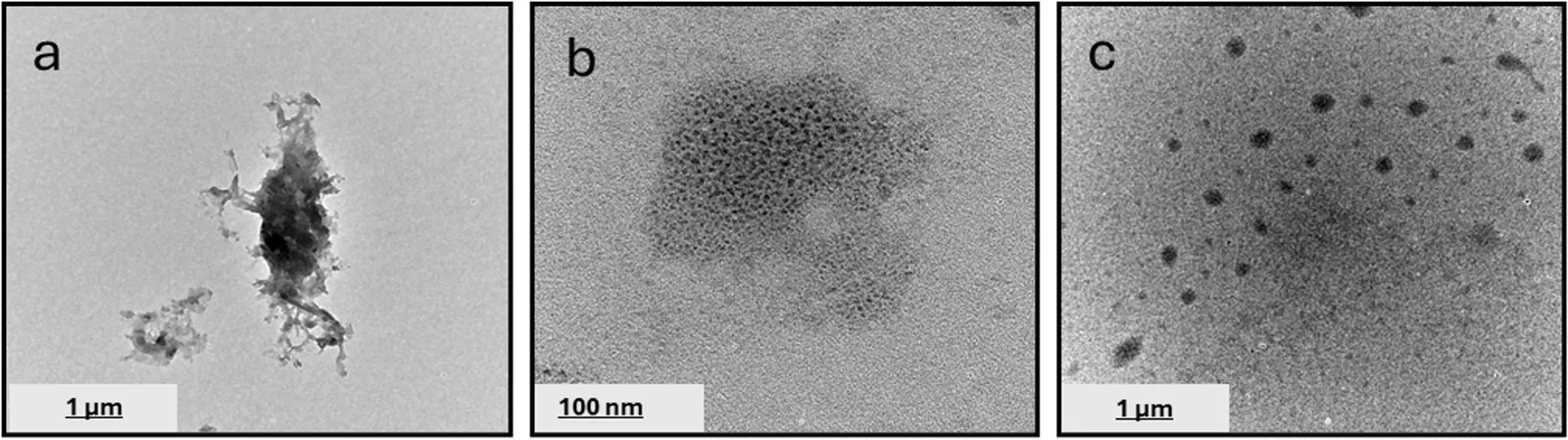
Transmission electron microscopy (TEM) images of (**a**) rosemary essential oil, (**b**) blank formulation (gum Arabic + Ag-NPs), and (**c**) rosemary essential oil nanoparticles (Ros-Es-NPs)

TEM images of gum Arabic and *Ros-Es-NPs* (Fig. 3b & c) revealed that they are spherical in shape and Nano sized, whereas the native essential oil (Fig. 3a) exhibited an amorphous shape and larger particle size, indicating the absence of nanoscale organization. In contrast, the TEM micrographs of gum Arabic and Ros-ES-NPs (Fig. 3b and c) revealed a well-defined spherical morphology with nanoscale dimensions, confirming successful nanoparticle formation. The transformation from an amorphous structure to a spherical nanoscale architecture following nano-formulation demonstrates the effective encapsulation of the essential oil within the gum Arabic matrix and supports the formation of a stable nano-system (Abdelaziz et al. 2024; Elbehery et al. 2025).

### Scanning Electron Microscopy (SEM)

**Fig. 4:**
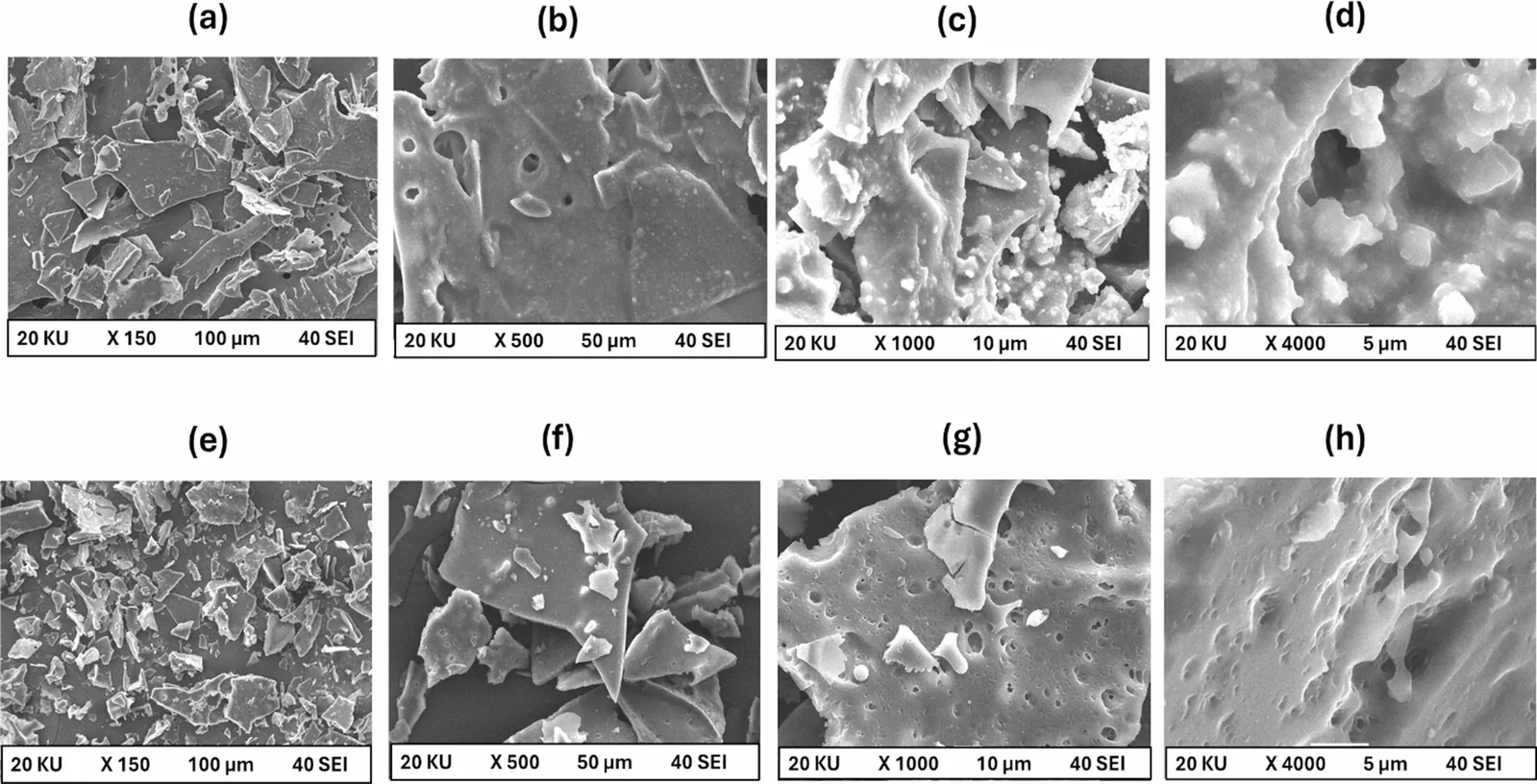
Micrographs of (**a**–**d**) blank formulation (gum Arabic + Ag-NPs) and (**e**–**h**) rosemary essential oil nanoparticles (Ros-Es-NPs) at different magnifications: 150×, 500×, 1000×, and 4000×. Scale bars are included in each image

Morphological examination of the fine powder of blank gum Arabic with Ag nanoparticles at different magnifications (Fig. 4a–d) showed a rocky texture with an outcropping exterior surface. In contrast, the addition of essential oil to the blank resulted in an exterior surface that became rocky and featured numerous holes and dents (Fig. 4e–h). The surface appeared highly irregular and porous, with numerous cavities, holes, and surface indentations, indicating disruption of the compact structure. These structural alterations suggest successful incorporation and interaction of the essential oil within the Arabic gum matrix, leading to the formation of a more porous and heterogeneous surface, which may enhance oil entrapment, release behavior, and overall nano-formulation stability (Eghbalifam et al. 2020).

### Thermogravimetric Analysis (TGA)

**Fig. 5:**
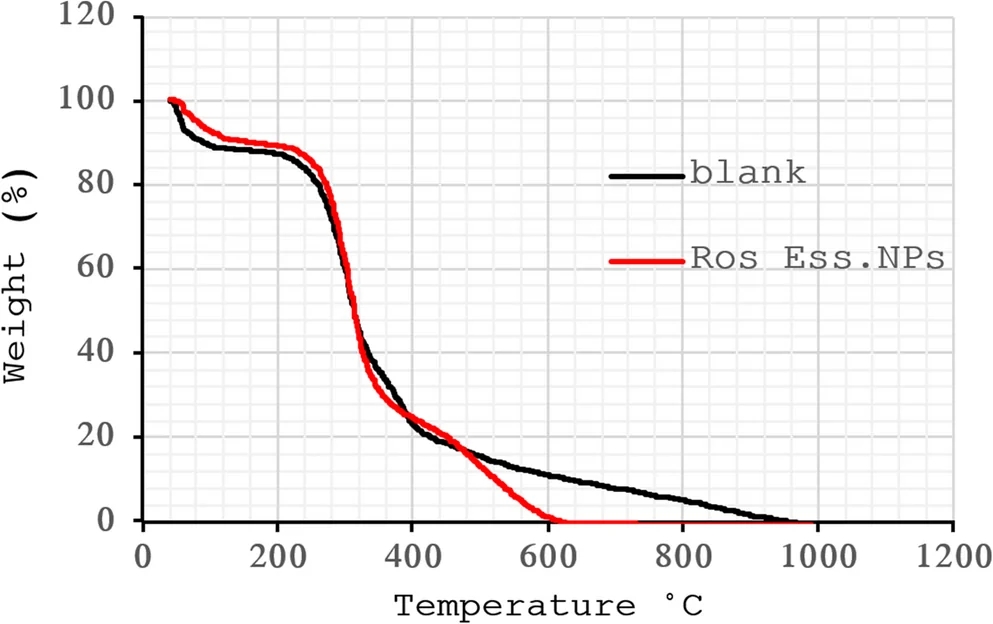
Thermogravimetric analysis (TGA) of gum Arabic with silver nanoparticles (blank) and rosemary essential oil nanoparticles (Ros-Es-NPs)

Figure 5 displays the thermal stability of gum Arabic with silver nanoparticles (black spectrum) and the Ros-Es-NPs (red spectrum). The thermographs for both the blank and the Ros-Es-NPs were obtained in air, with a heating rate of 10 °C/min using TGA. The results showed that the native blank exhibited a weight loss of approximately 45% at 328 °C, which increased to 67% at 427 °C. In comparison, the addition of Ros-Es to the blank nanoparticles resulted in a greater weight loss of 62.9% at 376 °C and 88% at 629 °C. This suggests that the Ros-Es-NPs are more heat-sensitive than the blank, likely because Ros-Es are inherently more reactive to heat than the polymers associated with Ag nanoparticles. Figure 4a–d) showed a rocky texture with an outcropping exterior surface. In contrast, the addition of essential oil to the blank resulted in an exterior surface that became rocky and featured numerous holes and dents (Fig. 4e–h).

### Particle size distribution

The surface charge of particles (zeta potential), polydispersity index (PDI), and particle size are critical parameters influencing the stability and effectiveness of nanomedicines in drug delivery systems. Table 1 presents the particle size (in nm) of the produced essential oil nanoparticles, which was found to be less than 100 nm. The particle size distribution of the different components is presented to provide a clearer comparison of the system. The prepared Ros-Es-NPs exhibited an average particle size of 83.9 nm, confirming their nanoscale range. In comparison, the native Ros-Es showed a much larger particle size (989.7 nm), while silver nanoparticles, gum Arabic, and the blank sample exhibited sizes of 433 nm, 134 nm, and 82.8 nm, respectively. These results indicate a significant reduction in particle size upon nano-formulation, along with improved dispersion relative to the native components.

**Table 1:** Polydispersity index (pdi), zeta potential and diameter of gum Arabic, silver-NPs, Blank, Ros-Es, and Ros-Es-NPs

	Gum Arabic	Silver	blank	Ros- Es.	Ros- Es-NPs
Pdi	0.665	0.480	0. 794	0.178	0.533
Zeta potential	-21.1	­ 31.1	­ 19.2	­ 5.87	­ 29.7
Z-Average (d.nm)	134.4	433.7	82.88	989.7	83.94

The PDI values were 0.66 for gum Arabic, 0.48 for silver nanoparticles, 0.794 for the blank, 0.178 for Ros-Es, and 0.533 for Ros-Es-NPs, indicating heterogeneous size distributions. Ros-Es demonstrated a narrow distribution (PDI = 0.178), whereas the blank and Ros-Es-NPs exhibited moderate to high polydispersity, suggesting a broader yet acceptable particle size distribution for such systems (Ahmed et al., 2021). The zeta potential values were measured as − 21.1 mV for gum Arabic, − 31.1 mV for silver nanoparticles, -19.2 for the blank, and + 5.87 mV and − 29.7 mV for both Ros-Es and Ros-Es-NPs respectively. Zeta potential values with an absolute magnitude above ± 30 mV suggest strong electrostatic stability, indicating that the formulated nanoparticles are likely to resist rapid coagulation or flocculation (Schulz et al. 2005).

### Cell viability

**Fig. 6:**
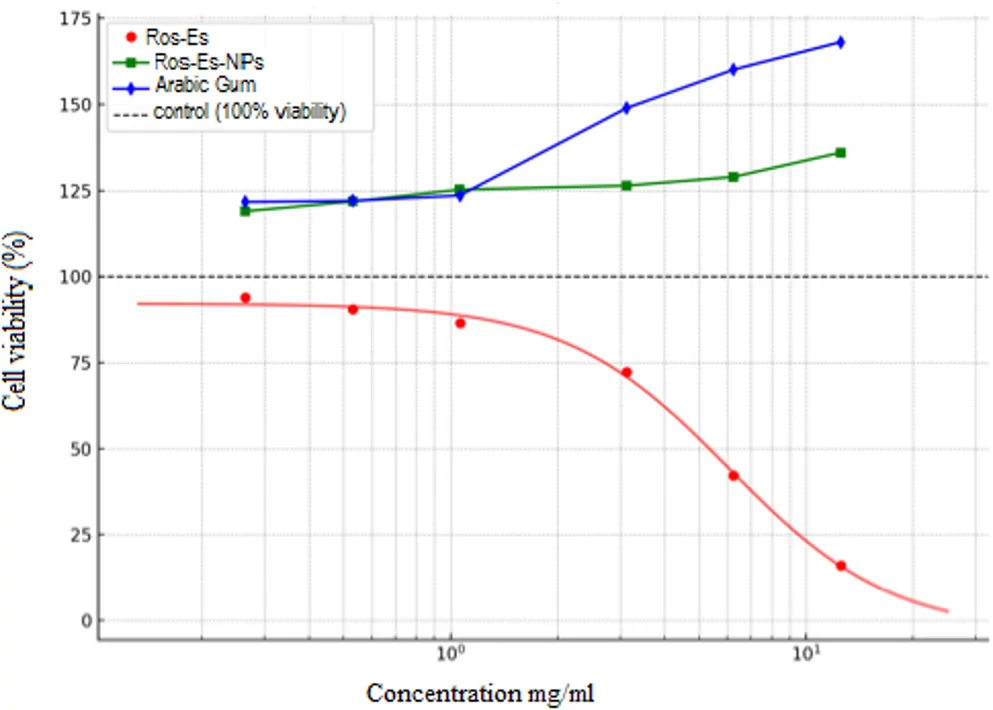
Effect of rosemary essential oil (Ros-Es), rosemary essential oil nanoparticles (Ros-Es-NPs), and gum Arabic on the viability of human lung fibroblast cells (Wi-38), determined by MTT assay. Data are expressed as percentage viability relative to untreated control cells (set at 100%). Values above 100% possibly due to high metabolic activity. Results represent mean ± standard deviation of triplicate experiments

Ros-ES exhibited a clear dose-dependent cytotoxic effect on the human lung fibroblast cell line (Wi-38), with an IC50 of approximately 6.06 mg/ml, indicating substantial toxicity at higher concentrations (Fig. 6).

In contrast, Ros-ES-NPs demonstrated no detectable cytotoxicity within the tested concentration range and showed apparent increases in cell viability, reaching approximately 136%. Similarly, Arabic gum exhibited markedly elevated viability values, up to approximately 168% (Table 2).

However, these values exceeding 100% should be interpreted with caution. The MTT assay measures mitochondrial metabolic activity rather than direct cell number; therefore, increased absorbance may reflect enhanced cellular metabolism rather than true proliferation. In addition, potential interactions between test compounds—particularly nanoparticles, essential oil constituents, and surfactants such as Tween 80—and the MTT assay system may influence formazan formation or optical density readings, leading to overestimation of viability.

Such observations are consistent with previous reports demonstrating that essential oils may exhibit dose-dependent cytotoxicity in normal cell lines (Bakkali et al. 2008), while nano-formulations can reduce toxicity and improve biocompatibility (Pascual-Mathey et al. 2022). Moreover, Arabic gum has been reported to possess antioxidant and prebiotic properties that may enhance cellular metabolic activity (Babiker et al. 2022), although assay-related effects cannot be excluded.

In the present study, concentrations above 12.5 mg/ml were not evaluated due to potential confounding factors, including micelle aggregation, medium turbidity, and alterations in pH and osmolarity, which may lead to non-specific or false-positive cytotoxicity results.

Overall, the findings suggest that nano-encapsulation significantly reduces the cytotoxicity of Ros-ES and improves its apparent biocompatibility. Nevertheless, further validation using complementary assays, such as lactate dehydrogenase (LDH) release, trypan blue exclusion, or fluorescence-based live/dead staining, is recommended to confirm these observations and distinguish between true cell proliferation and increased metabolic activity.

**Table 2:** Interpretation of cell viability results of Ros-Es, Ros-Es-NPs, and Arabic Gum on human lung fibroblast cell line (Wi-38)

Sample	Effect at 12.5 mg/ml	Notes
Ros-ES	≈ 16% viability → highly cytotoxic	IC50 ≈ 6.06 mg/ml
Ros-ES-Nps	≈ 136% viability →increased metabolic activity	No Toxicity observed
Arabic Gum	≈ 168% viability → high metabolic activity,	Potential bioactive effect

### The antibacterial activity

The present study demonstrates that rosemary essential oil – nanoparticles significantly enhances its antibacterial activity against both Gram-positive (*Streptococcus pneumoniae*) and Gram-negative *(Shigella flexneri* and *K. pneumoniae*) bacteria, alongside *C. albicans* as the fungal pathogen. It can be seen from Table 3, the superior performance of Ros-ES-NPs compared to free Ros-ES is evident from both inhibition percentages and estimated IC₅₀ values. This enhanced antibacterial activity is consistent with recent studies reporting that nano formulation of essential oils improves their stability, solubility, and bioavailability, thereby increasing their effectiveness against microbial pathogens (Eid et al. 2022; Gadallah et al. 2024; Al-Hayali and Atiyea 2024). The reduced particle size facilitates closer interaction with bacterial membranes, increasing permeability and promoting leakage of intracellular components. In addition, nano-delivery systems enable sustained release of active compounds, prolonging antimicrobial action (Salem et al. 2025).

**Table 3:** The antibacterial capacity (% inhibition) and estimated IC50 of Ros-ES and Ros-ES-NPs

Microorganism	Treatment	25 mg/mL (% inhibition)	12.5 mg/mL LSD (% inhibition)	IC50 (mg/mL)
*S. pneumoniae*	Ros-ES	68.75^b^±0.60	52.85^c^±0.97 12.29	≈ 11.0
	Ros-ES-NPs	80.73^a^±5.61	62.50^b^±4.05	< 12.5
*Shigella flexneri*	Ros-ES	76.47^b^±1.16	59.40^c^±0.90 4.47	≈ 10.5
	Ros-ES-NPs	87.13^a^±2.63	72.67^b^±3.68	< 12.5
*K. pneumoniae*	Ros-ES	55.16^bc^±0.09	44.68^c^±10.17 13.67	≈ 14.0
	Ros-ES-NPs	95.66^a^±9.74	64.17^b^±3.57	< 12.5
*C. albicans*	Ros-ES	81.85^a^±0.54	27.56^c^±3.48 6.63	≈ 19.0
	Ros-ES-NPs	60.39^b^±5.69	9.76^d^±0.91	≈ 10.5

*All experiments were performed in triplicate; all data are expressed as the mean ± SD. Means with different letters (at the same Colum) are significantly different at *p* ≤ 0.05 according to the Least Significant Difference (LSD) test. The group labeled “a” has the highest mean value and is significantly different from those labeled “b” and “c”. The selected concentration treatments were informed by prior well diffusion assays.

*IC50 values were estimated by linear interpolation between tested concentrations and should be interpreted with caution.

Specifically, at 12.5 mg/ml, pure Ros-ES inhibited *K. pneumoniae and Shigella flexneri* by 44.68 ± 10.17% and 59.40 ± 0.90%, respectively, while Ros-ES-NPs inhibited them by 64.17 ± 3.57% and 72.67 ± 3.68%, respectively. At 25 mg/ml, Ros-ES inhibited *K. pneumoniae and Shigella flexneri* by 55.16 ± 0.09% and 76.47 ± 1.16%, respectively, whereas Ros-ES-NPs showed inhibition rates of 95.66 ± 9.74% and 87.13 ± 2.63%, respectively. Against *Streptococcus pneumoniae*, at 12.5 mg/ml, Ros-ES showed 52.85 ± 0.97% inhibition, compared to 62.50 ± 4.05% for Ros-ES-NPs. At 25 mg/ml, Ros-ES inhibited by 68.75 ± 0.60%, while Ros-ES-NPs exhibited a higher inhibition rate of 80.73 ± 5.6%.

The strong antibacterial activity observed against *K. pneumoniae* is particularly noteworthy, as this organism is known for its intrinsic resistance mechanisms. Similar findings have been reported, where rosemary essential oil nanoparticle formulation Ros-Es-NPs exhibited significantly enhanced antibacterial activity compared to free Ros-ES, with reduced inhibitory concentrations (Gadallah et al. 2024). The increased efficacy is likely due to improved penetration through the bacterial outer membrane, where the nano formulation Ros-Es-NPs protects volatile bioactive compounds such as 1,8-cineole and α-pinene, preserving their antimicrobial activity and facilitating their delivery to bacterial outer membrane and enhanced interaction with cellular targets. Furthermore, Ros-Es-NPs may enhance penetration into biofilms, which are often associated with increased antimicrobial resistance (Salem et al. 2025).

On the contrary, Hassanzadazar et al. 2019 found that pure Ros-ES showed better inhibitory effects than prepared Ros-ES- Nano emulsion on all studied bacteria. The variation in antimicrobial activity between free rosemary essential oil and its nano-formulation can be explained by multiple interacting mechanisms related to compound availability, release kinetics, and microbial cell structure (Moghimi et al. 2016; Ahmed et al. 2025). In the nano-formulation, bioactive constituents of the essential oil are partially entrapped within the gum Arabic matrix and associated with surfactant molecules, which can temporarily limit their immediate interaction with microbial cell membranes (Hamed et al. 2024). While this encapsulation enhances stability and allows controlled release, it may reduce the initial concentration of freely available active molecules, particularly those responsible for rapid membrane disruption. In bacterial cells, especially Gram-negative species, the nano-sized particles facilitate closer contact with the outer membrane, improving permeability and enabling a sustained antimicrobial effect (Nazzaro et al. 2013). The presence of silver nanoparticles may further enhance this effect through membrane destabilization, oxidative stress induction, and interference with intracellular components, including proteins and DNA. These combined mechanisms explain the superior antibacterial performance of Ros-ES-NPs observed in the present study.

In contrast, the antifungal results against *C. albicans* revealed a different trend, where free Ros-ES demonstrated higher activity at higher concentration (81.85 ± 0.54) compared to the nanoparticle formulation Ros-Es-NPs (60.39 ± 5.69) which is consistent with Neves et al. 2019. This may be attributed to the complex structure of fungal cell walls, which contain chitin, β-glucans and mannoproteins that can limit nanoparticle (Ros-Es-NPs) penetration. The free Ros-ES components are extremely lipophilic and volatile compounds that can rapidly diffuse through the cell membrane of a fungus. This can cause immediate damage to the cell membrane and disrupt its metabolism. However, nanoparticle formulation Ros-Es-NPs can slow down this process due to its controlled release mechanism. As a result, a lesser immediate antifungal effect is seen compared to the free Ros-ES. This is also supported by the study carried out by Eid et al. (2022), where organism-dependent differences were seen for the antimicrobial response of nano-encapsulated essential oils. Additionally, the presence of gum Arabic may influence the release profile of active compounds, reducing their availability in antifungal applications. Similar variability in antifungal responses has been reported in previous studies (Gadallah et al. 2024).

It is important to note that the IC₅₀ values reported in this study were estimated based on a limited concentration range due to formulation constraints associated with gum Arabic viscosity. While this approach allows comparative evaluation, it does not replace standard MIC determination. Previous studies using broth microdilution methods have confirmed that nano formulated rosemary oil exhibits lower MIC values compared to free oil (Al-Hayali and Atiyea 2024). Therefore, further investigation using a broader concentration range is recommended.

### DNA fragmentation assay

Essential oils (Eos) and their active components can interact with microbial DNA either directly intercalation (Qu et al. 2019), fragmentation (Khan et al. 2017) or indirectly -ROS generation (Nazzaro et al. 2013), and enzyme inhibition (Helander et al. 1998). This contributed to their antimicrobial activity. Recent studies have further demonstrated that nanoparticle-based delivery systems can enhance these effects by improving intracellular delivery and amplifying oxidative stress-mediated damage (Salmani-Zarchi et al. 2024).

**Fig. 7:**
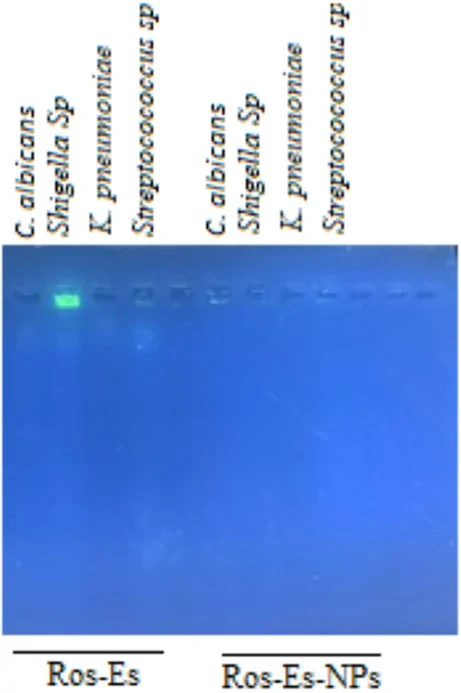
Agarose gel electrophoresis of genomic DNA isolated from *Candida albicans*, *Shigella flexneri*, *Klebsiella pneumoniae*, and *Streptococcus pneumoniae*. Lanes 1–4 correspond to samples treated with Ros-Es, and lanes 6–9 correspond to samples treated with Ros-Es-NPs

Agarose gel electrophoresis analysis (Fig. 7) revealed distinct differences in DNA integrity between treatment groups. In samples treated with ROS-ES, partial DNA fragmentation was observed, as indicated by smeared bands and the presence of lower molecular weight fragments. This pattern suggests DNA strand breakage, likely resulting from oxidative stress or nuclease-mediated degradation.

In contrast, samples treated with ROS-ES-NPs showed an absence of visible DNA bands on the agarose gel. This observation indicates a substantially higher degree of DNA disruption compared to the free essential oil. Nanoparticle-mediated antimicrobial activity has been associated with enhanced ROS generation and direct interaction with genetic material, which can lead to severe DNA damage and inhibition of replication processes (Ali et al., 2023).

Importantly, all samples were processed under identical experimental and DNA extraction conditions, and a consistent pattern was observed within each treatment group across all tested microorganisms. ROS-ES-treated samples consistently exhibited partial DNA fragmentation, whereas ROS-ES-NP-treated samples consistently showed absence of detectable DNA bands. This reproducibility strongly supports a treatment-dependent effect rather than random experimental variability or extraction-related artifacts.

Nevertheless, it is important to acknowledge that agarose gel electrophoresis has inherent limitations in detecting highly fragmented or chemically modified DNA. The absence of visible bands may reflect extensive degradation into fragments too small to be resolved; however, alternative explanations such as reduced DNA recovery or technical limitations cannot be completely excluded. Therefore, while the findings strongly suggest enhanced DNA disruption by ROS-ES-NPs, they do not conclusively demonstrate complete DNA elimination.

Further confirmatory analyses, including comet assay, quantitative PCR (qPCR), or fluorescence-based DNA integrity assays, would provide deeper insight into the extent and mechanism of DNA damage, and are recommended for future investigations.

## Conclusions

The findings of this study highlight the superior antimicrobial potential of rosemary essential oil when formulated as nanoparticles, while showing no cytotoxic effects on human lung fibroblast cells. The enhanced activity of ROS-ES-NPs appears to be mediated through multiple mechanisms, including improved cellular uptake, increased oxidative stress, and more DNA damage.

The consistent and treatment-specific DNA damage patterns observed across all tested microorganisms strongly support a genuine biological effect of the nanoparticle formulation. These results align with recent advances in antimicrobial nanotechnology, which emphasize the role of nanoparticles in overcoming microbial resistance through multi-target mechanisms.

Overall, ROS-ES-NPs represent a promising antimicrobial platform with potential applications in combating resistant microbial strains. However, further molecular-level investigations are required to fully elucidate the underlying mechanisms of DNA damage and validate these findings using complementary analytical approaches.

## Supplementary Information

Below is the link to the electronic supplementary material.

Supplementary Material 1.

## Data Availability

Data is provided within the manuscript and supplementary information files. Also, the raw data supporting the findings of this study are provided in the supplementary file in the submission system.
