# Supplementary material for: Rosemary essential oil nanoparticles enhance antimicrobial activity and induce bacterial DNA disruption
Source: Int Microbiol. 2026 May 13;29(7):1149–59. doi: 10.1007/s10123-026-00815-2 (PMC13653882; doi:10.1007/s10123-026-00815-2)
Supplement: Supplementary file 1 [file 10123_2026_815_MOESM1_ESM.pdf]

Size Distribution Report by Intensity
v2.2

Malvern

Sample Details

Sample Name: 3 size 4

SOP Name: Size Water 0.3

General Notes:

**File Name:**	3203.del	**Dispersant Name:**	Water
**Record Number:**	1036	**Dispersant RI:**	1.330
**Material RI:**	0.10	**Viscosity (cP):**	0.8872
**Material Absorbtion:**	0.300	**Measurement Date and Time:**	Wednesday, May 8, 2024 12:...

System

**Temperature (°C):**	25.0	**Duration Used (s):**	10
**Count Rate (kcps):**	255.1	**Measurement Position (mm):**	5.50
**Cell Description:**	Clear disposable zeta cell	**Attenuator:**	11

Results

			Size (d.nm):	% Intensity:	St Dev (d.n...
**Z-Average (d.nm):**	82.88	**Peak 1:**	1605	40.6	1063
**PdI:**	0.794	**Peak 2:**	120.4	36.5	42.75
**Intercept:**	0.817	**Peak 3:**	22.54	22.9	7.247

Result quality : **Refer to quality report**

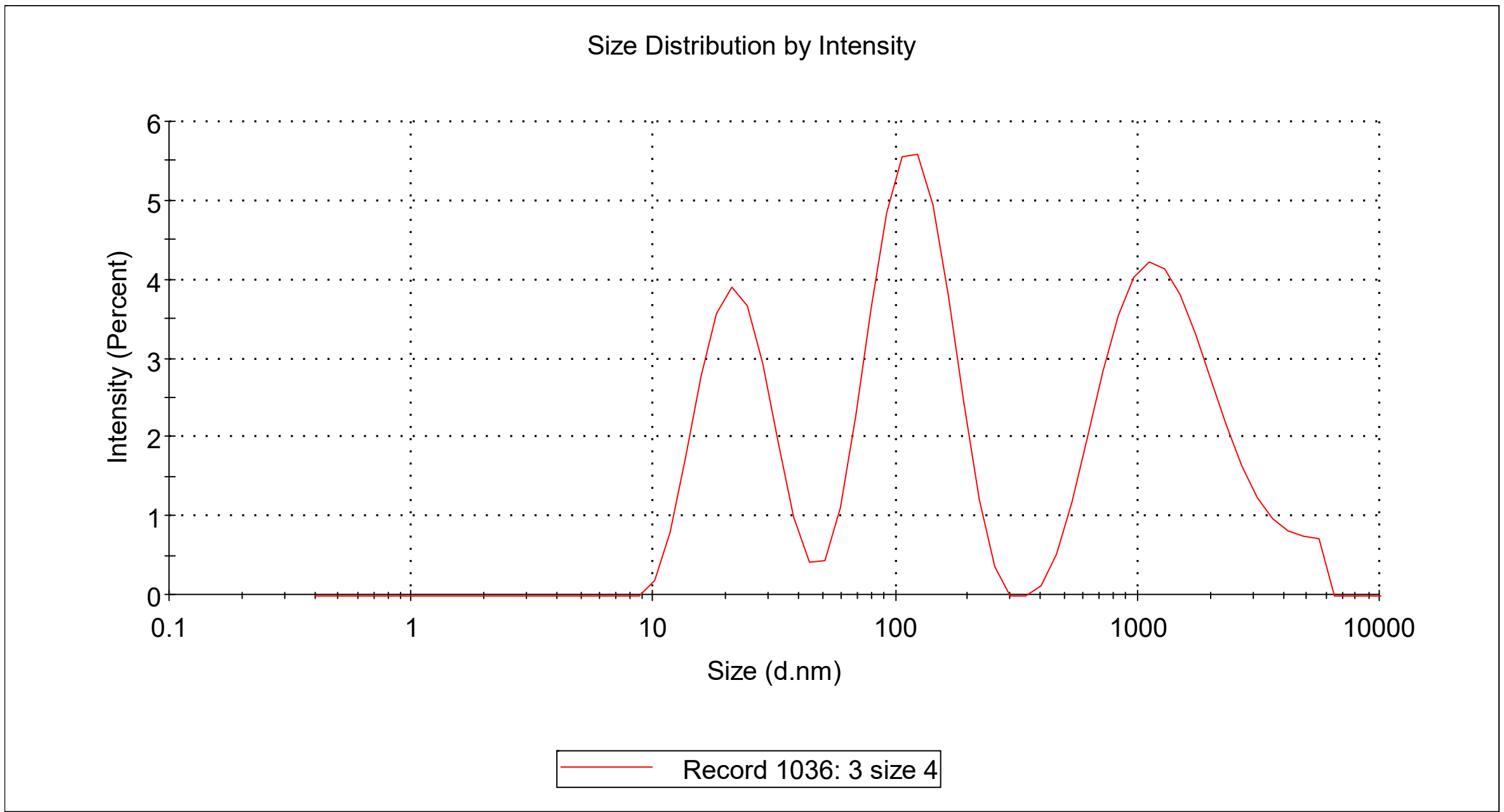

Zeta Potential Report

v2.3

Malvern Instruments Ltd - © Copyright 2008

Sample Details

Sample Name: 3 3

SOP Name: RI 1.3 water.sop

General Notes:

**File Name:**	3203.del	**Dispersant Name:**	Water
**Record Number:**	1039	**Dispersant RI:**	1.330
**Date and Time:**	Wednesday, May 8, 2024 12:41:4...	**Viscosity (cP):**	0.8872
		**Dispersant Dielectric Constant:**	78.5

System

**Temperature (°C):**	25.0	**Zeta Runs:**	12
**Count Rate (kcps):**	228.7	**Measurement Position (mm):**	2.00
**Cell Description:**	Clear disposable zeta cell	**Attenuator:**	11

Results

			**Mean (mV)**	**Area (%)**	**St Dev (mV)**
**Zeta Potential (mV):**	-19.2	**Peak 1:**	-19.2	100.0	6.51
**Zeta Deviation (mV):**	6.51	**Peak 2:**	0.00	0.0	0.00
**Conductivity (mS/cm):**	0.0310	**Peak 3:**	0.00	0.0	0.00
**Result quality :**	Good				

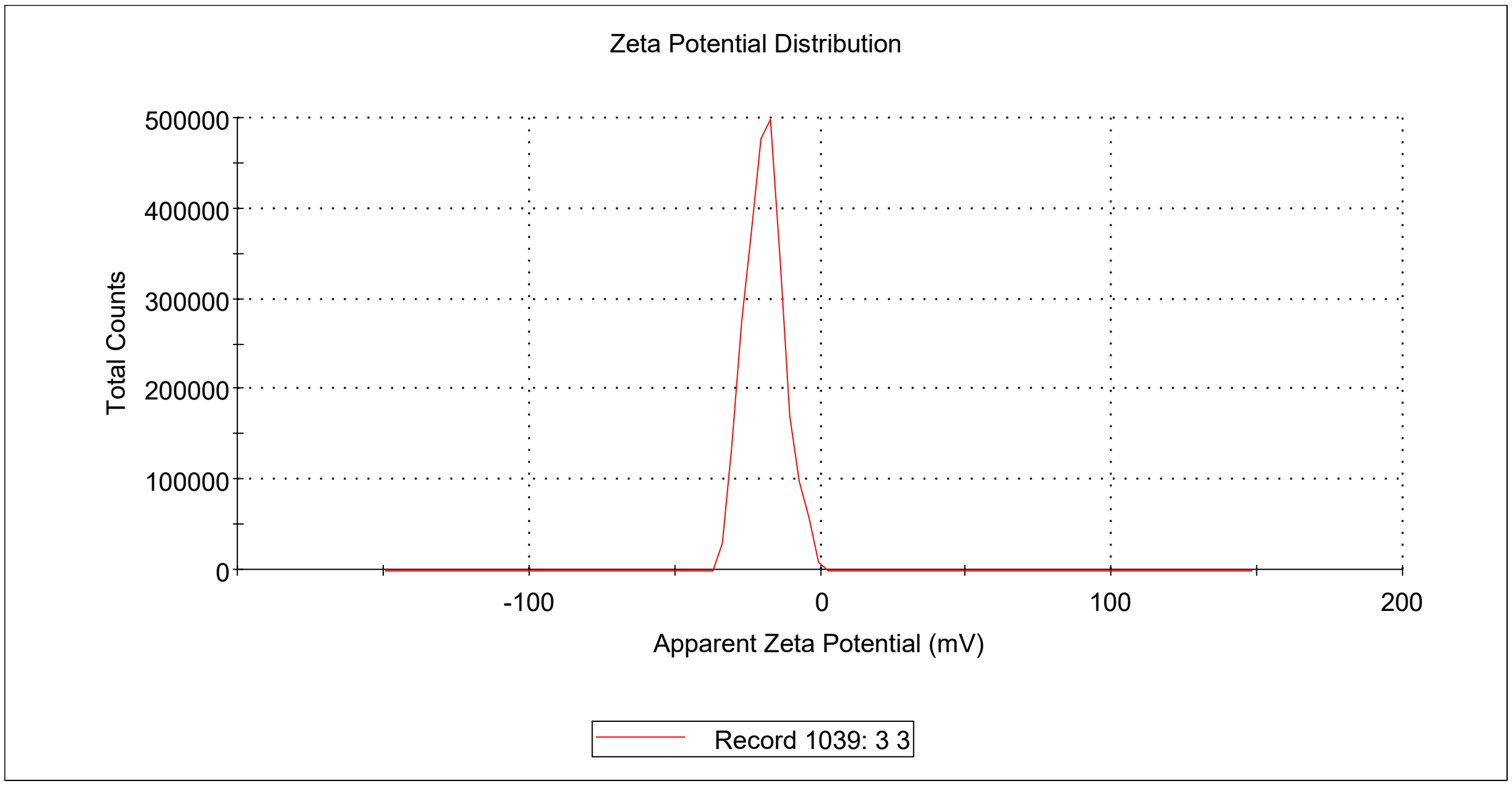

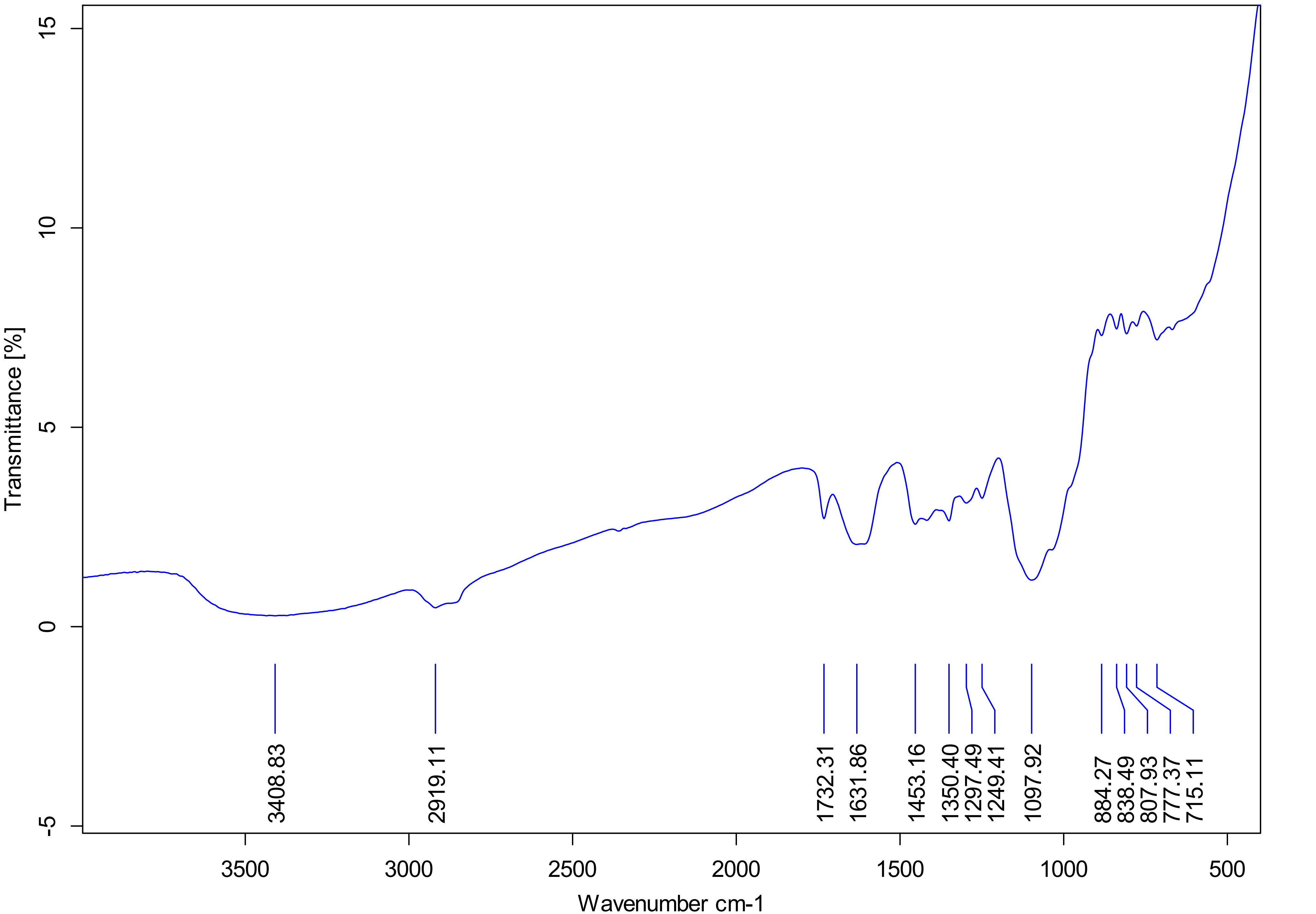
Transmittance [%]
Wavenumber cm-1
15
10
5
0
-5
3500
3000
2500
2000
1500
1000
500
3408.83
2919.11
1732.31
1631.86
1453.16
1350.40
1297.49
1249.41
1097.92
884.27
838.49
807.93
777.37
715.11

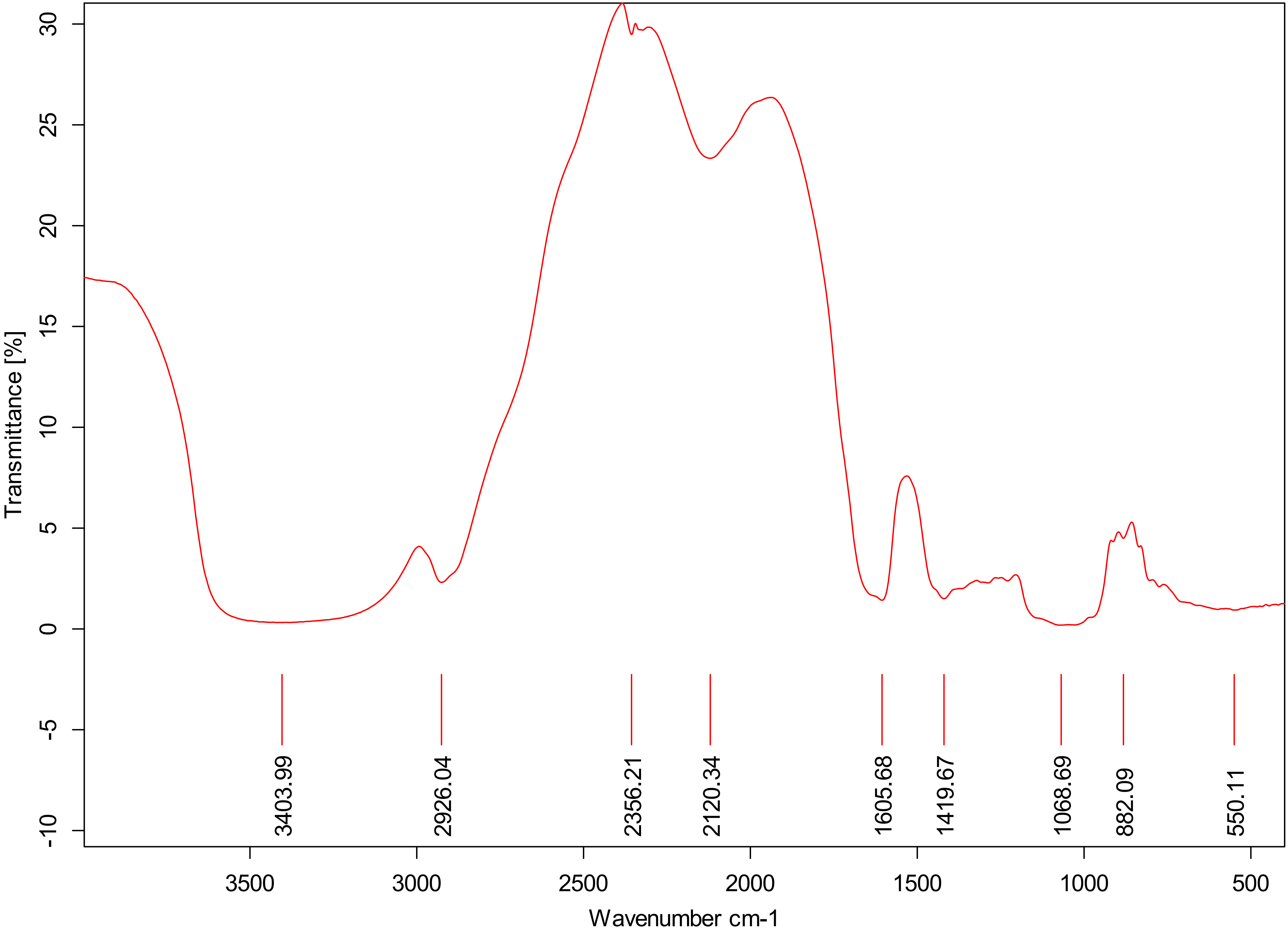
Transmittance [%]
30
25
20
15
10
5
0
-5
-10
3403.99
2926.04
2356.21
2120.34
1605.68
1419.67
1068.69
882.09
550.11
3500
3000
2500
2000
1500
1000
500
Wavenumber cm-1

Zeta Potential Report
v2.3

Malvern Instruments Ltd - © Copyright 2008

Sample Details

Sample Name: 1 2

SOP Name: RI 1.3 water.sop

General Notes:

**File Name:**	3203.del	**Dispersant Name:**	Water
**Record Number:**	1024	**Dispersant RI:**	1.330
**Date and Time:**	Wednesday, May 8, 2024 12:06:2...	**Viscosity (cP):**	0.8872
		**Dispersant Dielectric Constant:**	78.5

System

**Temperature (°C):**	25.0	**Zeta Runs:**	12
**Count Rate (kcps):**	262.9	**Measurement Position (mm):**	2.00
**Cell Description:**	Clear disposable zeta cell	**Attenuator:**	10

Results

			**Mean (mV)**	**Area (%)**	**St Dev (mV)**
**Zeta Potential (mV):**	-21.1	**Peak 1:**	-21.1	100.0	5.86
**Zeta Deviation (mV):**	5.86	**Peak 2:**	0.00	0.0	0.00
**Conductivity (mS/cm):**	0.0410	**Peak 3:**	0.00	0.0	0.00
**Result quality :**	Good				

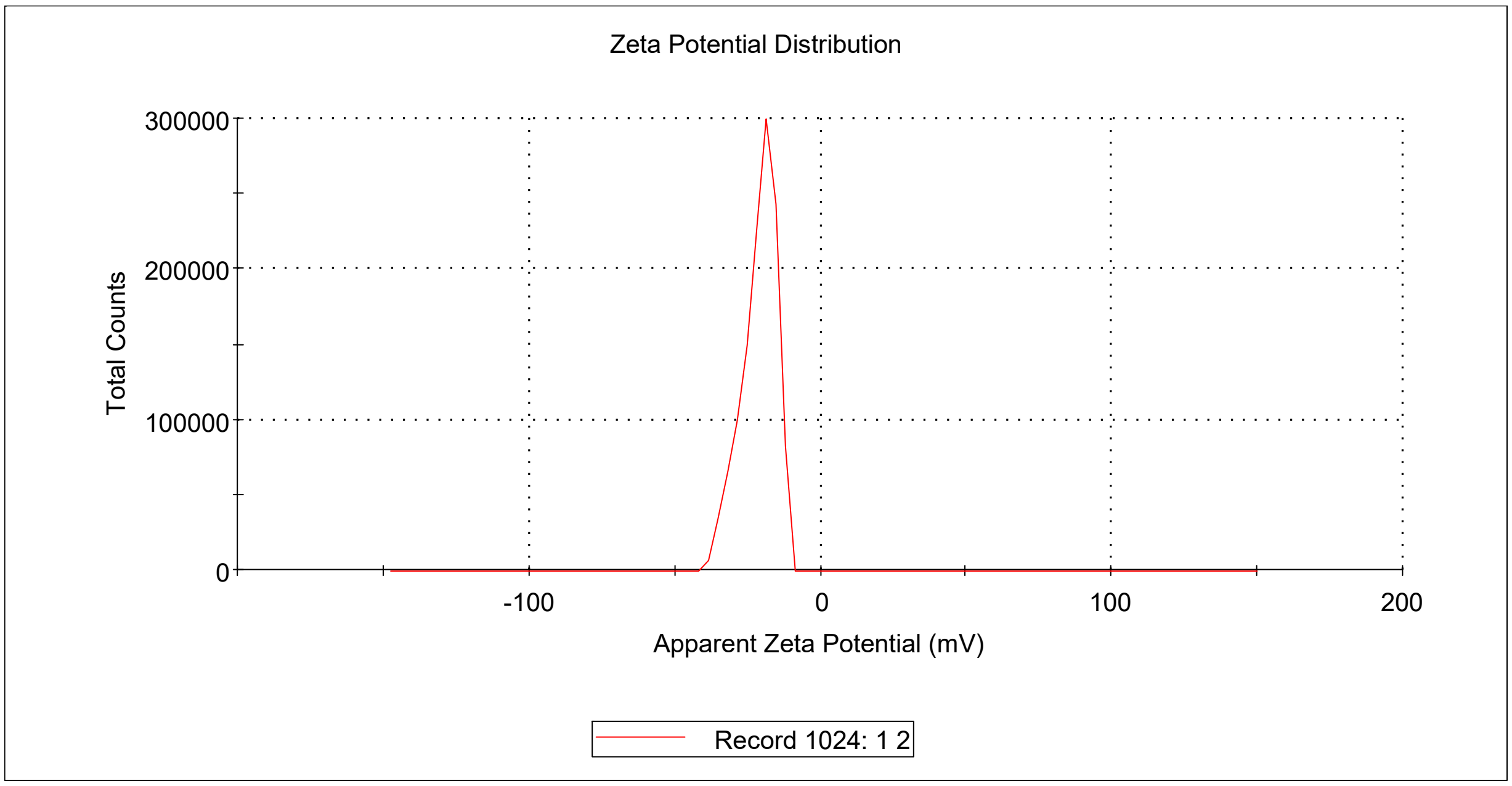

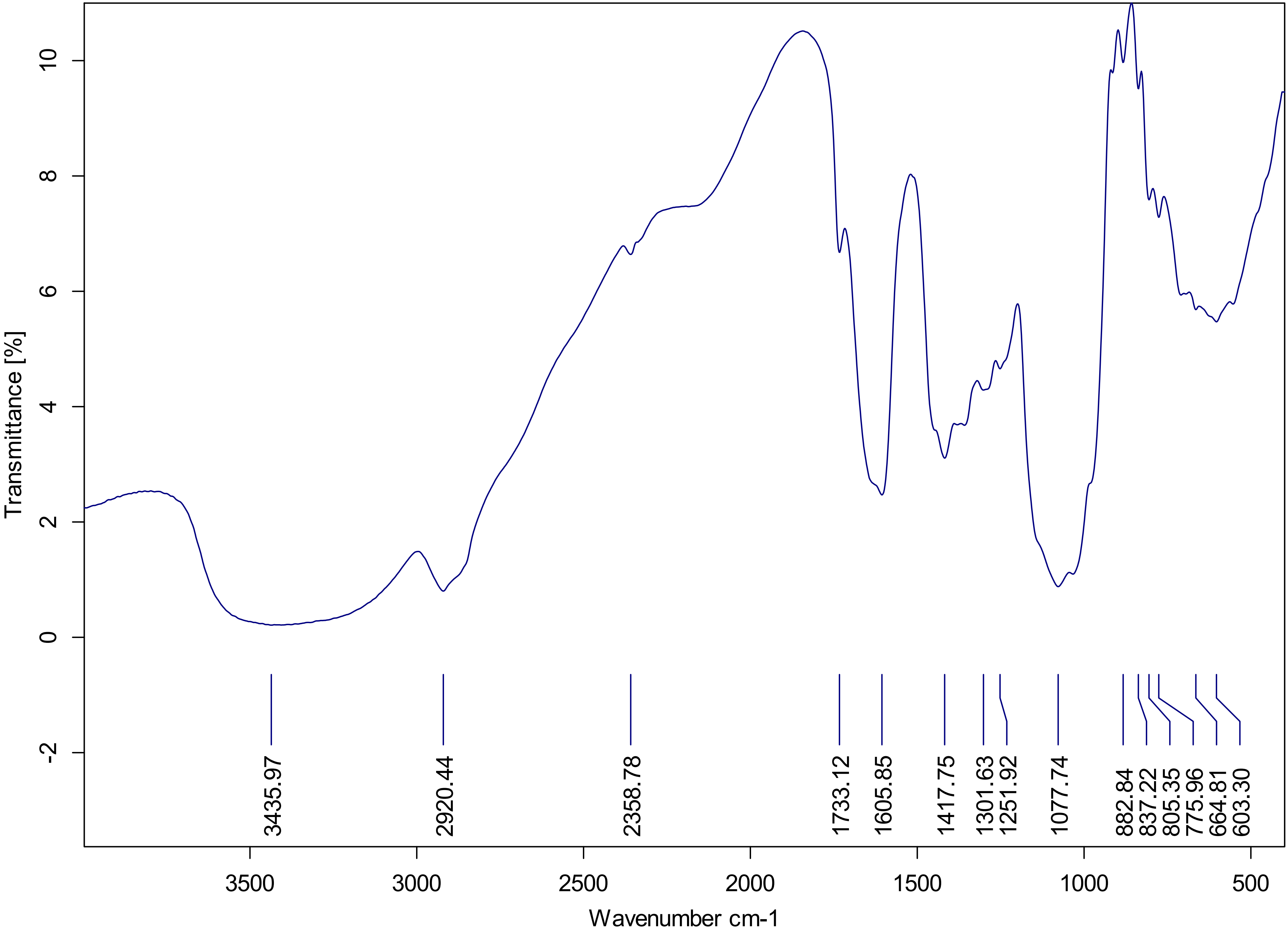
Transmittance [%]
Wavenumber cm-1
10
8
6
4
2
0
-2
3500
3000
2500
2000
1500
1000
500
3435.97
2920.44
2358.78
1733.12
1605.85
1417.75
1301.63
1251.92
1077.74
882.84
837.22
805.35
775.96
664.81
603.30

Zeta Potential Report

v2.3

Malvern Instruments Ltd - © Copyright 2008

Sample Details

Sample Name: 4 2

SOP Name: RI 1.3 water.sop

General Notes:

**File Name:**	3203.del	**Dispersant Name:**	Water
**Record Number:**	1044	**Dispersant RI:**	1.330
**Date and Time:**	Wednesday, May 8, 2024 12:58:0...	**Viscosity (cP):**	0.8872
		**Dispersant Dielectric Constant:**	78.5

System

**Temperature (°C):**	25.0	**Zeta Runs:**	12
**Count Rate (kcps):**	1179.1	**Measurement Position (mm):**	2.00
**Cell Description:**	Clear disposable zeta cell	**Attenuator:**	11

Results

			**Mean (mV)**	**Area (%)**	**St Dev (mV)**
**Zeta Potential (mV):**	-29.7	**Peak 1:**	-29.7	100.0	7.83
**Zeta Deviation (mV):**	7.83	**Peak 2:**	0.00	0.0	0.00
**Conductivity (mS/cm):**	0.0526	**Peak 3:**	0.00	0.0	0.00
**Result quality :**	Good				

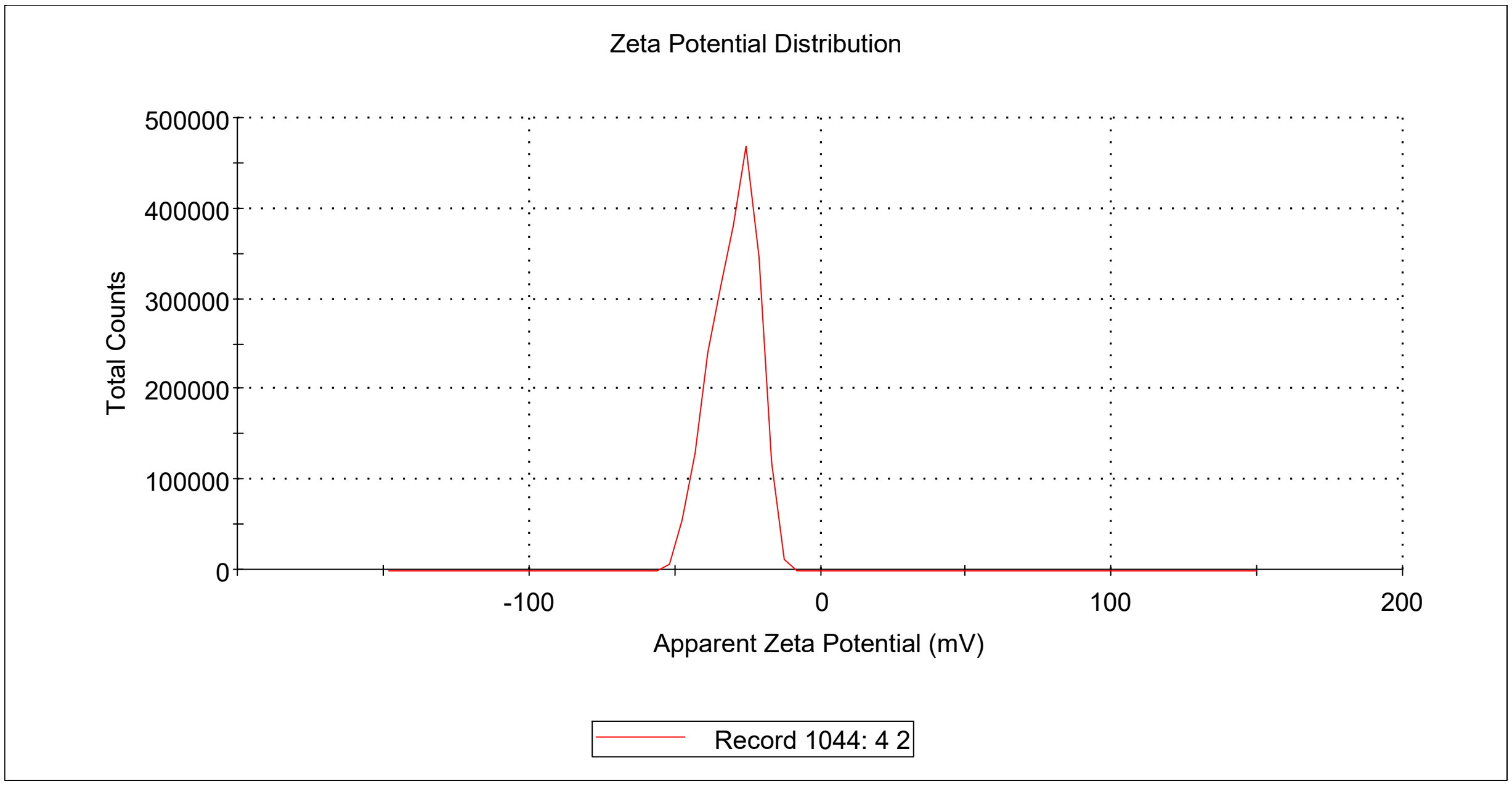

SHIMADZU

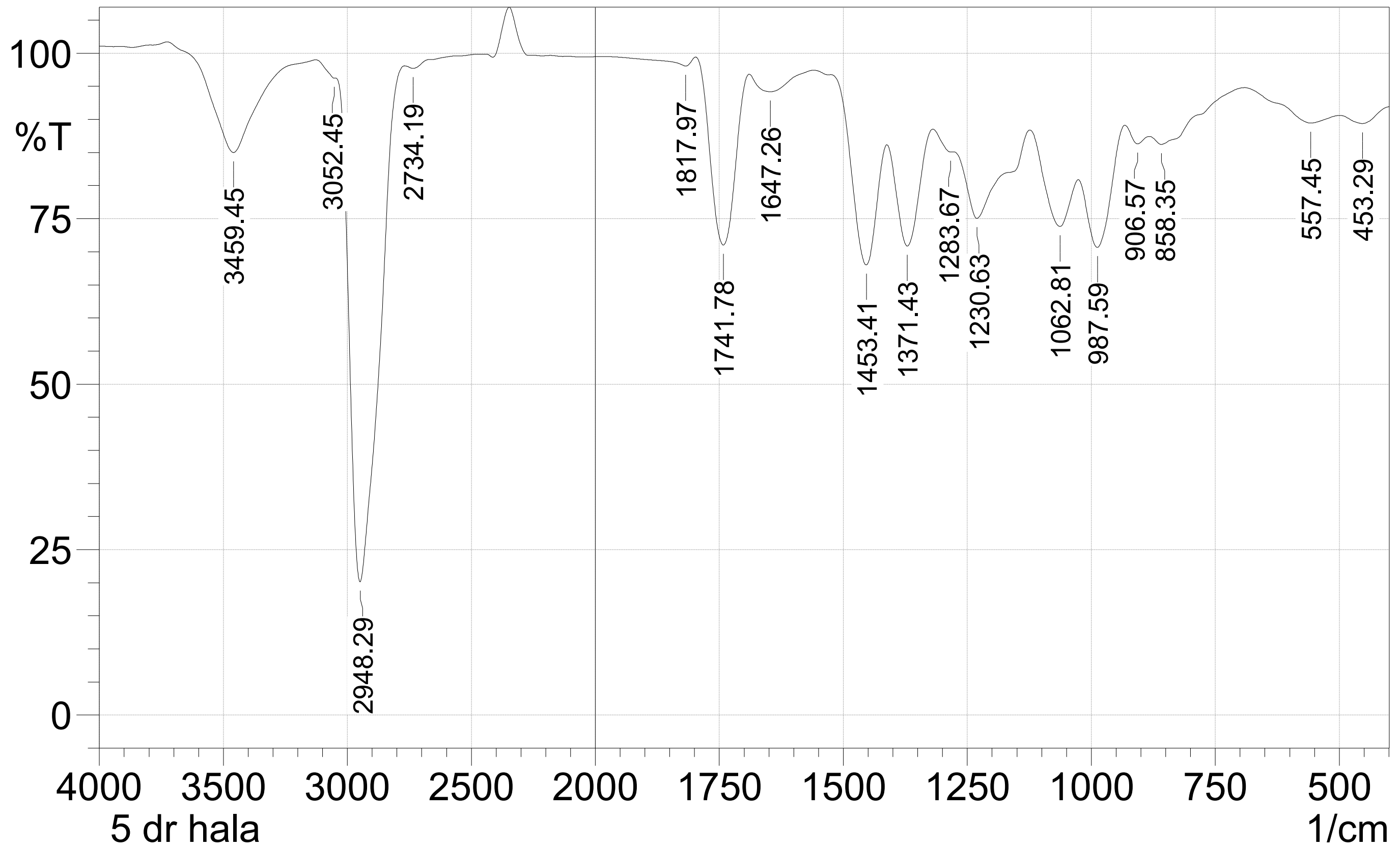

No.	Peak	Intensity	Corr. Intensity	Base (H)	Base (L)	Area	Corr. Area
1	453.29	89.384	1.831	498.62	399.28	4.387	0.45
2	557.45	89.443	2.441	690.54	499.58	7.318	1.013
3	858.35	86.229	2.124	882.46	691.5	8.418	0.752
4	906.57	86.31	1.923	931.65	883.43	2.857	0.239
5	987.59	70.675	13.619	1026.16	932.61	10.234	3.613
6	1062.81	73.839	9.83	1123.57	1027.13	9.804	2.763
7	1230.63	75.061	11.059	1277.88	1124.54	14.016	4.545
8	1283.67	85.067	0.447	1319.35	1278.85	2.565	0.072
9	1371.43	70.861	16.357	1411.94	1320.32	9.438	4.06
10	1453.41	68.027	21.985	1524.78	1412.9	10.281	5.871
11	1647.26	94.188	2.843	1689.7	1560.46	2.498	0.869
12	1741.78	71.018	27.053	1796.75	1690.66	8.064	7.179
13	1817.97	98.056	1.378	1979.03	1797.72	0.803	0.377
14	2734.19	97.709	0.721	2769.87	2667.64	0.787	0.154
15	2948.29	20.128	76.777	3046.67	2770.84	72.499	69.059
16	3052.45	96.228	0.186	3126.71	3047.63	0.835	0.031
17	3459.45	85.006	15.512	3726.6	3127.68	13.729	14.666

Comment;
5 dr hala

Date/Time; 18/12/2002 11:56:51
No. of Scans;
Resolution;
Apodization;
User; user

Zeta Potential Report
v2.3

Malvern Instruments Ltd - © Copyright 2008

Sample Details

Sample Name: 5 1

SOP Name: RI 1.4 aceton.sop

General Notes:

**File Name:**	3203.del	**Dispersant Name:**	Acetone
**Record Number:**	1052	**Dispersant RI:**	1.356
**Date and Time:**	Wednesday, May 8, 2024 1:19:28...	**Viscosity (cP):**	0.3111
		**Dispersant Dielectric Constant:**	20.7

System

**Temperature (°C):**	25.0	**Zeta Runs:**	12
**Count Rate (kcps):**	207.5	**Measurement Position (mm):**	2.00
**Cell Description:**	Clear disposable zeta cell	**Attenuator:**	7

Results

			**Mean (mV)**	**Area (%)**	**St Dev (mV)**
**Zeta Potential (mV):**	-5.87	**Peak 1:**	-5.87	100.0	7.10
**Zeta Deviation (mV):**	7.10	**Peak 2:**	0.00	0.0	0.00
**Conductivity (mS/cm):**	0.00902	**Peak 3:**	0.00	0.0	0.00
**Result quality :**	Good				

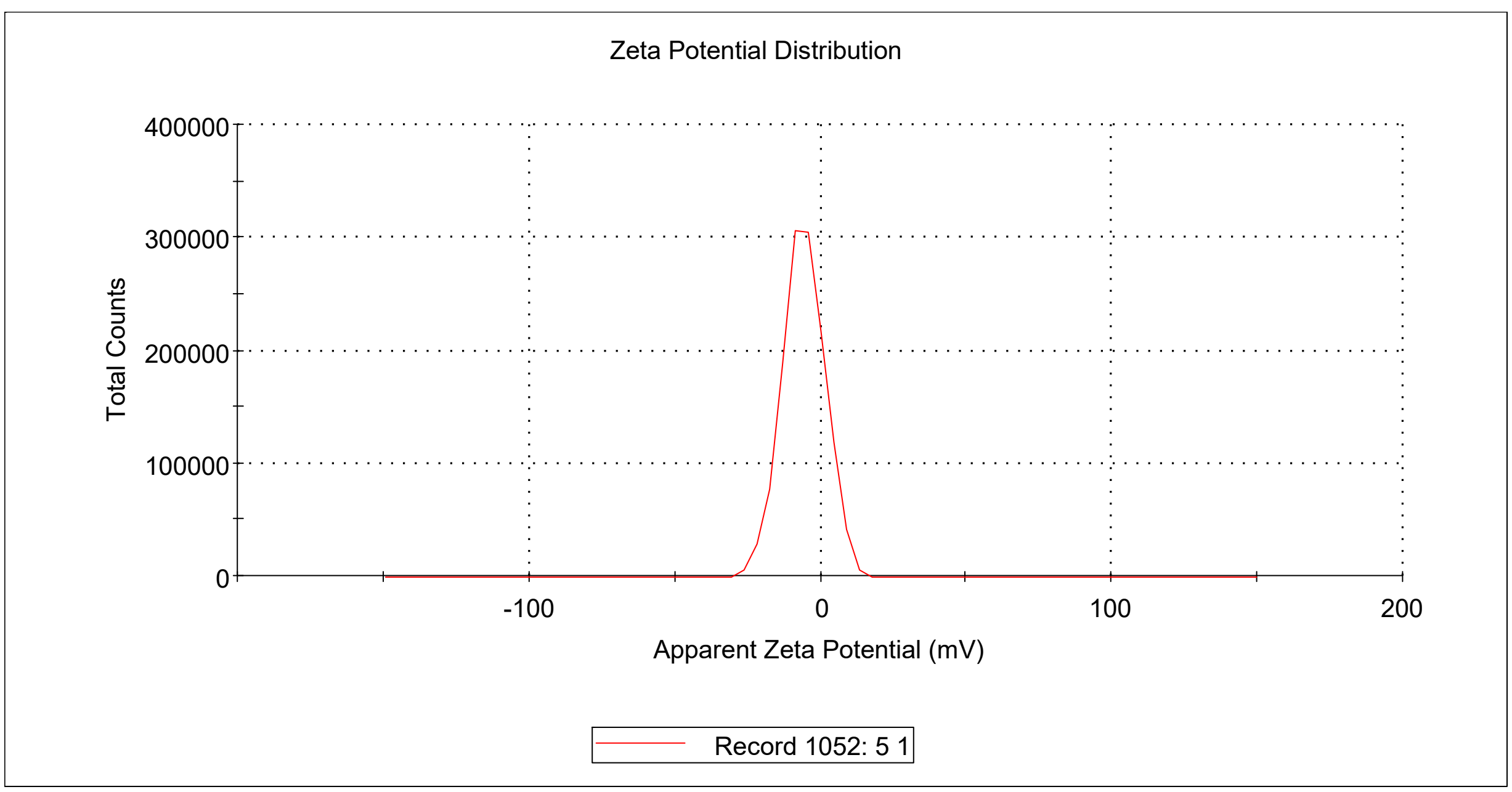

Zeta Potential Report

v2.3

Malvern Instruments Ltd - © Copyright 2008

Sample Details

Sample Name: 2 3

SOP Name: Zeta 0.113 water.sop

General Notes:

**File Name:**	3203.del	**Dispersant Name:**	Water
**Record Number:**	1032	**Dispersant RI:**	1.330
**Date and Time:**	Wednesday, May 8, 2024 12:25:0...	**Viscosity (cP):**	0.8872
		**Dispersant Dielectric Constant:**	78.5

System

**Temperature (°C):**	25.0	**Zeta Runs:**	17
**Count Rate (kcps):**	169.9	**Measurement Position (mm):**	2.00
**Cell Description:**	Clear disposable zeta cell	**Attenuator:**	11

Results

			Mean (mV)	Area (%)	St Dev (mV)
**Zeta Potential (mV):**	-31.1	**Peak 1:**	-31.1	100.0	7.52
**Zeta Deviation (mV):**	7.52	**Peak 2:**	0.00	0.0	0.00
**Conductivity (mS/cm):**	0.0523	**Peak 3:**	0.00	0.0	0.00
**Result quality :**	Good				

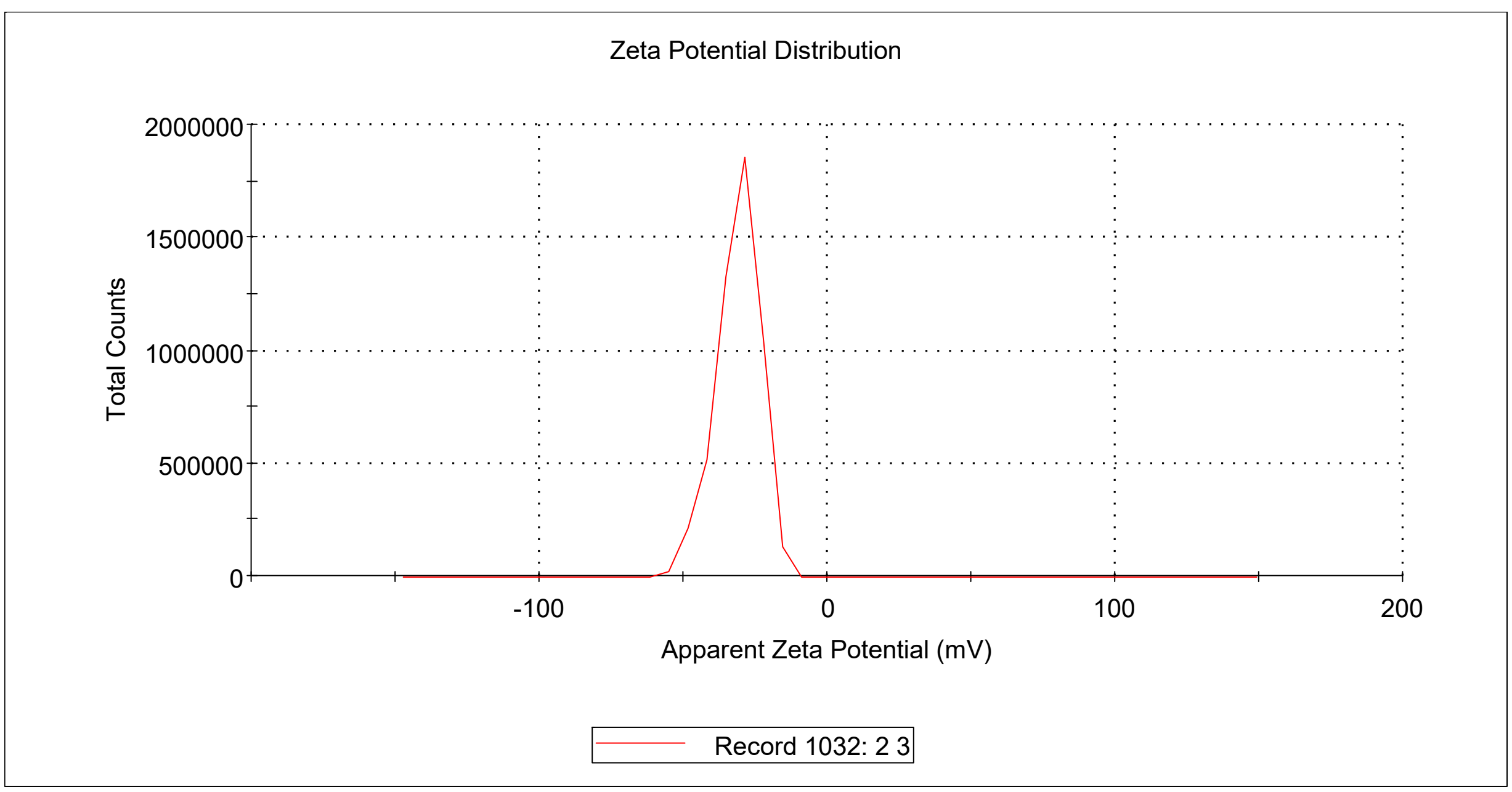

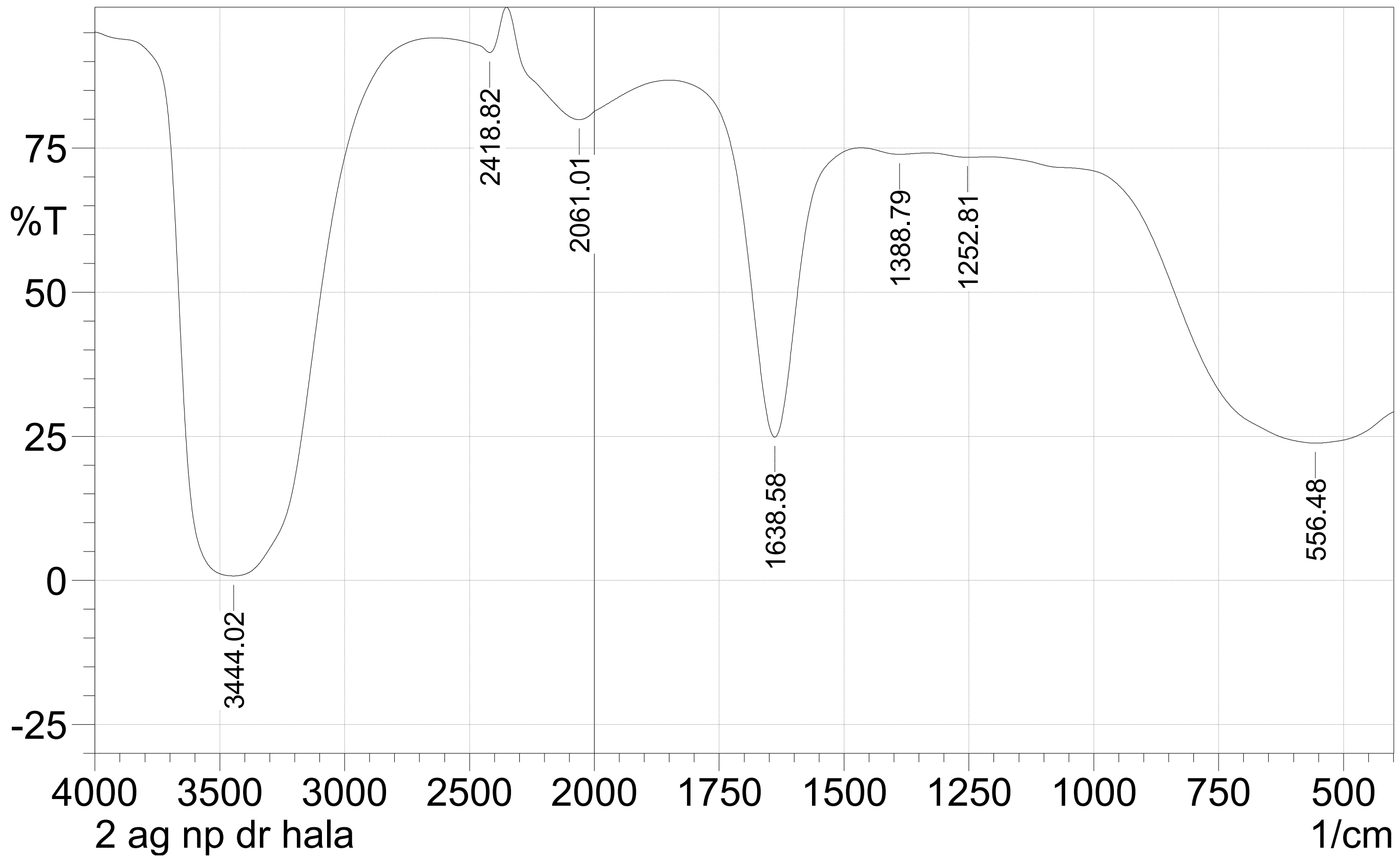

No.	Peak	Intensity	Corr. Intensity	Base (H)	Base (L)	Area	Corr. Area
1	556.48	23.82	14.04	1209.41	399.28	301.32	52.93
2	1252.81	73.41	0.31	1330.93	1210.37	16.01	0.11
3	1388.79	73.92	0.62	1464.98	1331.89	17.18	0.25
4	1638.58	24.84	55.51	1848.83	1465.95	81.04	45.55
5	2061.01	79.92	12.2	2351.3	1849.8	35.64	19.59
6	2418.82	91.58	6.63	2636.78	2352.27	7.94	3.87
7	3444.02	0.78	93.95	4000.5	2637.74	770.25	737.61

Comment;
2 ag np dr hala

Date/Time; 18/12/2002 11:51:11
No. of Scans;
Resolution;
Apodization;
User; user

Size Distribution Report by Intensity

v2.2

Malvern

Sample Details

Sample Name: 1 size 3

SOP Name: Size Water 0.3

General Notes:

**File Name:**	3203.del	**Dispersant Name:**	Water
**Record Number:**	1021	**Dispersant RI:**	1.330
**Material RI:**	0.10	**Viscosity (cP):**	0.8872
**Material Absorbtion:**	0.300	**Measurement Date and Time:**	Wednesday, May 8, 2024 12:...

System

**Temperature (°C):**	25.0	**Duration Used (s):**	60
**Count Rate (kcps):**	40.1	**Measurement Position (mm):**	5.50
**Cell Description:**	Clear disposable zeta cell	**Attenuator:**	9

Results

			**Size (d.nm):**	**% Intensity:**	**St Dev (d.n...**
**Z-Average (d.nm):**	134.4	**Peak 1:**	355.6	66.3	242.8
**PdI:**	0.665	**Peak 2:**	32.17	26.0	7.858
**Intercept:**	0.779	**Peak 3:**	4996	7.7	613.3

Result quality : **Refer to quality report**

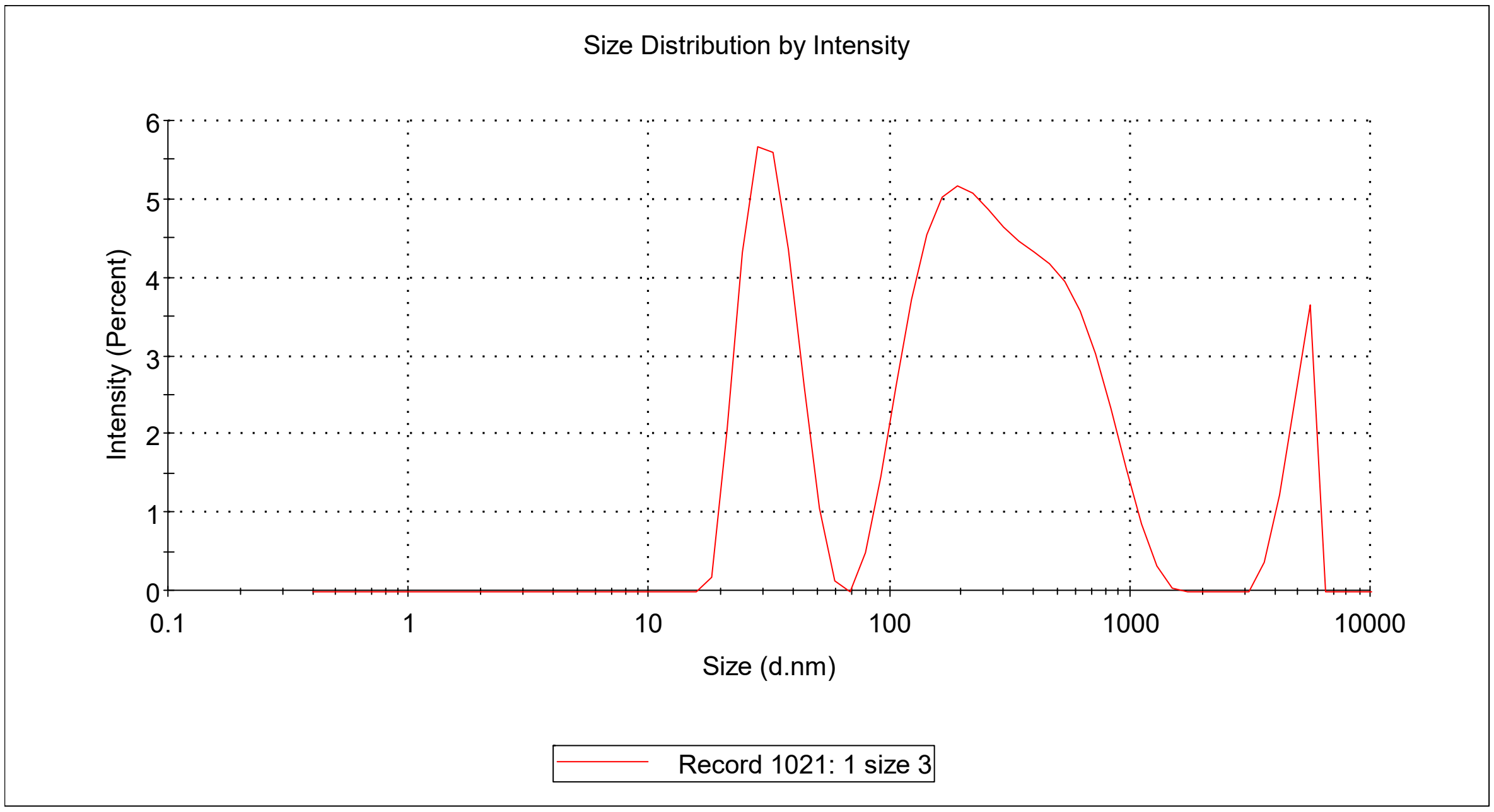

Size Distribution Report by Intensity

v2.2

Malvern

Sample Details

**Sample Name:**	4 size 3
**SOP Name:**	Size Water 0.3
**General Notes:**	

**File Name:**	3203.del	**Dispersant Name:**	Water
**Record Number:**	1042	**Dispersant RI:**	1.330
**Material RI:**	0.10	**Viscosity (cP):**	0.8872
**Material Absorbtion:**	0.300	**Measurement Date and Time:**	Wednesday, May 8, 2024 12:...

System

**Temperature (°C):**	25.0	**Duration Used (s):**	70
**Count Rate (kcps):**	94.3	**Measurement Position (mm):**	5.50
**Cell Description:**	Clear disposable zeta cell	**Attenuator:**	10

Results

			**Size (d.nm):**	**% Intensity:**	**St Dev (d.n...**
**Z-Average (d.nm):**	83.94	**Peak 1:**	186.3	57.1	118.7
**PdI:**	0.533	**Peak 2:**	34.10	32.7	13.09
**Intercept:**	0.900	**Peak 3:**	5010	7.6	606.0

Result quality : **Refer to quality report**

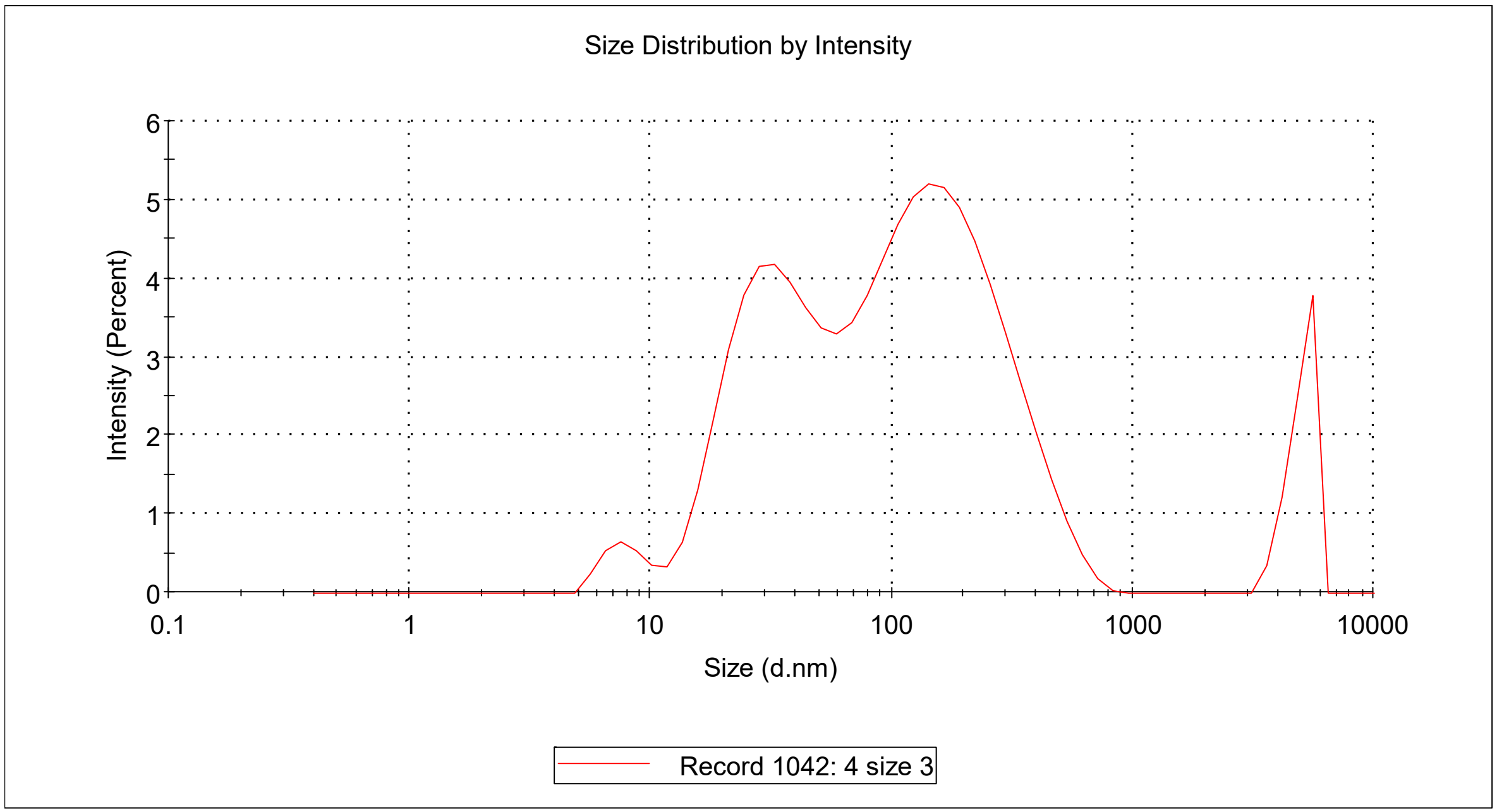

Size Distribution Report by Intensity

v2.2

Sample Details

**Sample Name:**	5 size 3
**SOP Name:**	Size 0.3 aceton.sop
**General Notes:**	

**File Name:**	3203.del	**Dispersant Name:**	Acetone
**Record Number:**	1051	**Dispersant RI:**	1.356
**Material RI:**	1.10	**Viscosity (cP):**	0.3111
**Material Absorbtion:**	0.300	**Measurement Date and Time:**	Wednesday, May 8, 2024 1:1...

System

**Temperature (°C):**	25.0	**Duration Used (s):**	70
**Count Rate (kcps):**	266.5	**Measurement Position (mm):**	5.50
**Cell Description:**	Clear disposable zeta cell	**Attenuator:**	7

Results

			Size (d.nm):	% Intensity:	St Dev (d.n...
**Z-Average (d.nm):**	989.7	**Peak 1:**	1073	97.8	426.8
**PdI:**	0.178	**Peak 2:**	5131	2.2	507.7
**Intercept:**	0.934	**Peak 3:**	0.000	0.0	0.000

Result quality : **Good**

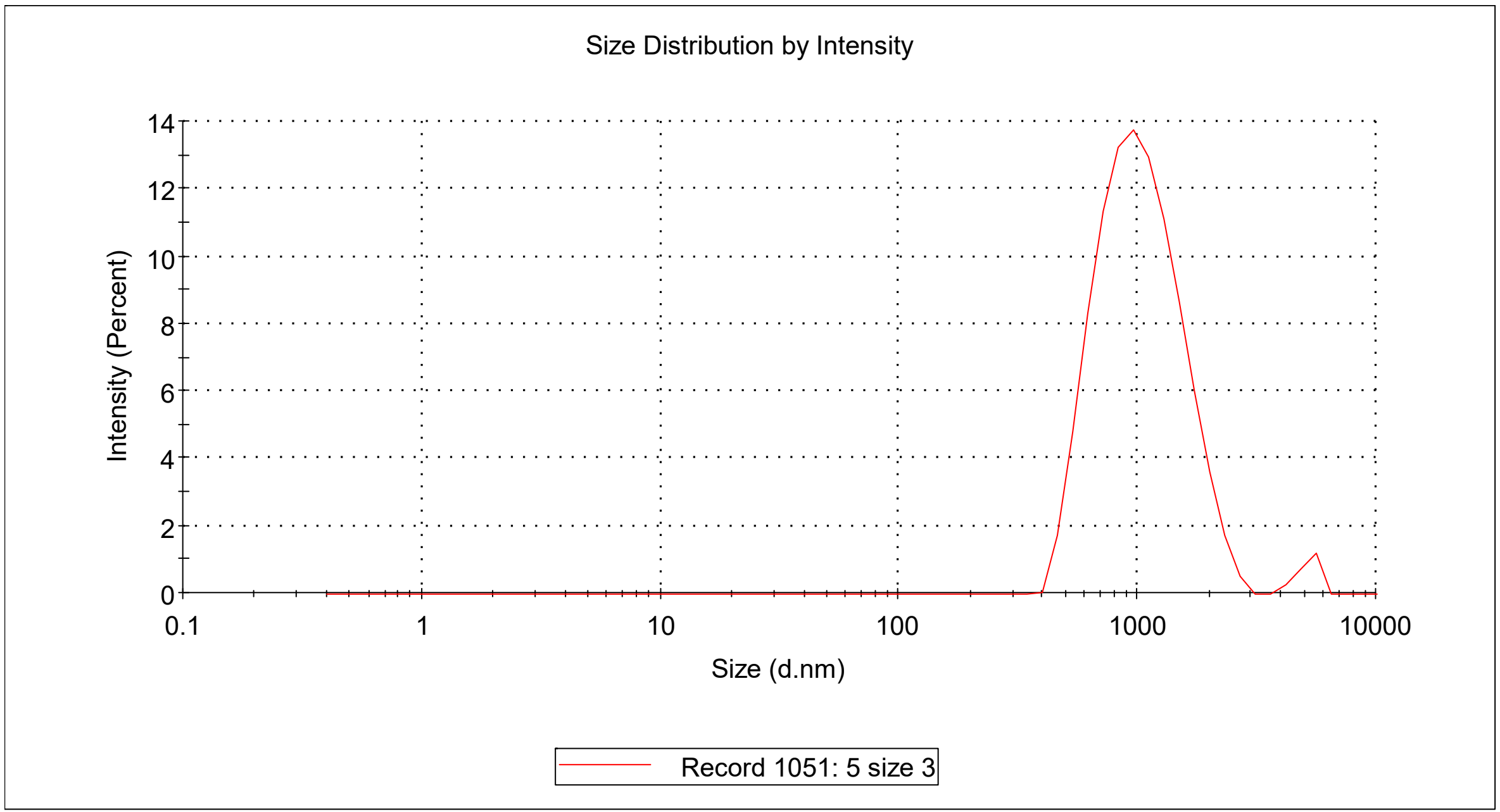

Size Distribution Report by Intensity

v2.2

Sample Details

Sample Name: 2 size 3

SOP Name: Size Water 0.3

General Notes:

**File Name:**	3203.del	**Dispersant Name:**	Water
**Record Number:**	1028	**Dispersant RI:**	1.330
**Material RI:**	0.10	**Viscosity (cP):**	0.8872
**Material Absorbtion:**	0.300	**Measurement Date and Time:**	Wednesday, May 8, 2024 12:...

System

**Temperature (°C):**	25.0	**Duration Used (s):**	60
**Count Rate (kcps):**	210.8	**Measurement Position (mm):**	5.50
**Cell Description:**	Clear disposable zeta cell	**Attenuator:**	11

Results

			Size (d.nm):	% Intensity:	St Dev (d.n...
**Z-Average (d.nm):**	433.7	**Peak 1:**	519.6	63.7	165.0
**PdI:**	0.480	**Peak 2:**	153.4	36.3	49.22
**Intercept:**	0.813	**Peak 3:**	0.000	0.0	0.000

Result quality : **Refer to quality report**

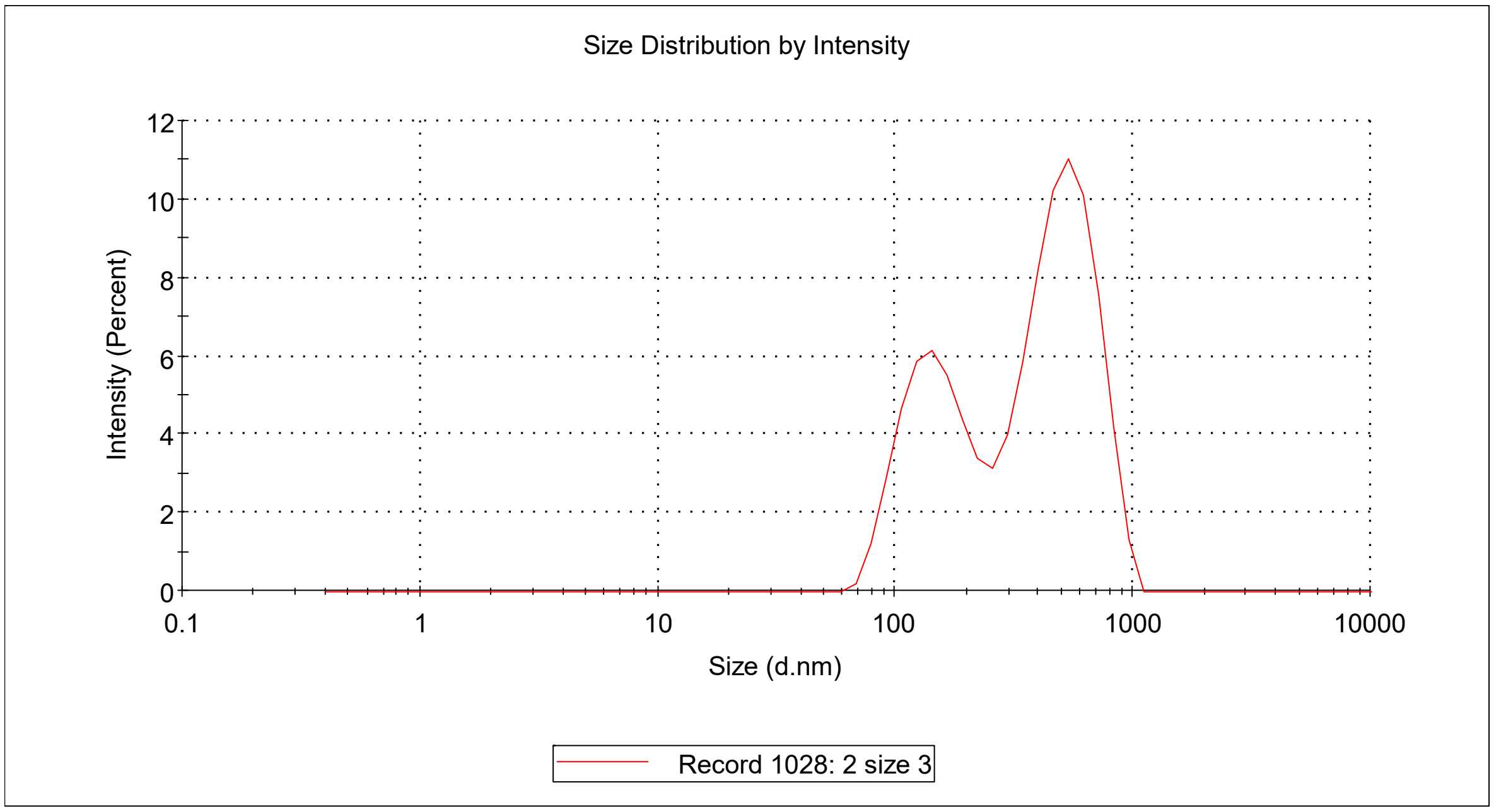

Sample: 3 DR.HALA
Size: 2.8683 mg

Comment: 10C/MIN

SDT

File: F:\SARAH\2024\25-6-2024\3 DR.HALA.UA
Operator: SARAH
Run Date: 25-Jun-2024 10:49

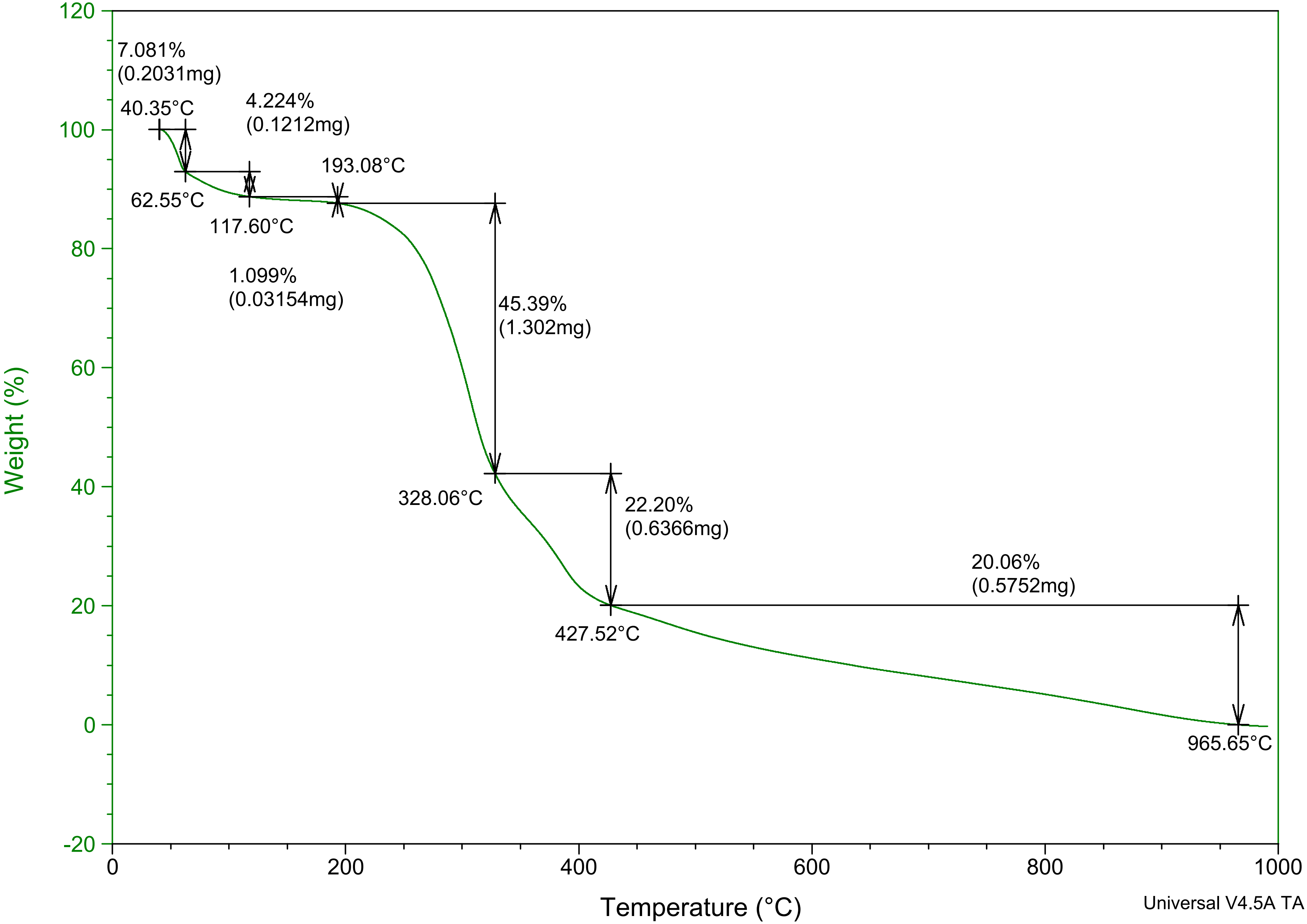

Universal V4.5A TA Instruments

Sample: 4 DR.HALA
Size: 2.4306 mg

Comment: 10C/MIN

SDT

File: F:\SARAH\2024\7-7-2024\4 DR.HALA.UA
Operator: SARAH
Run Date: 07-Jul-2024 10:39

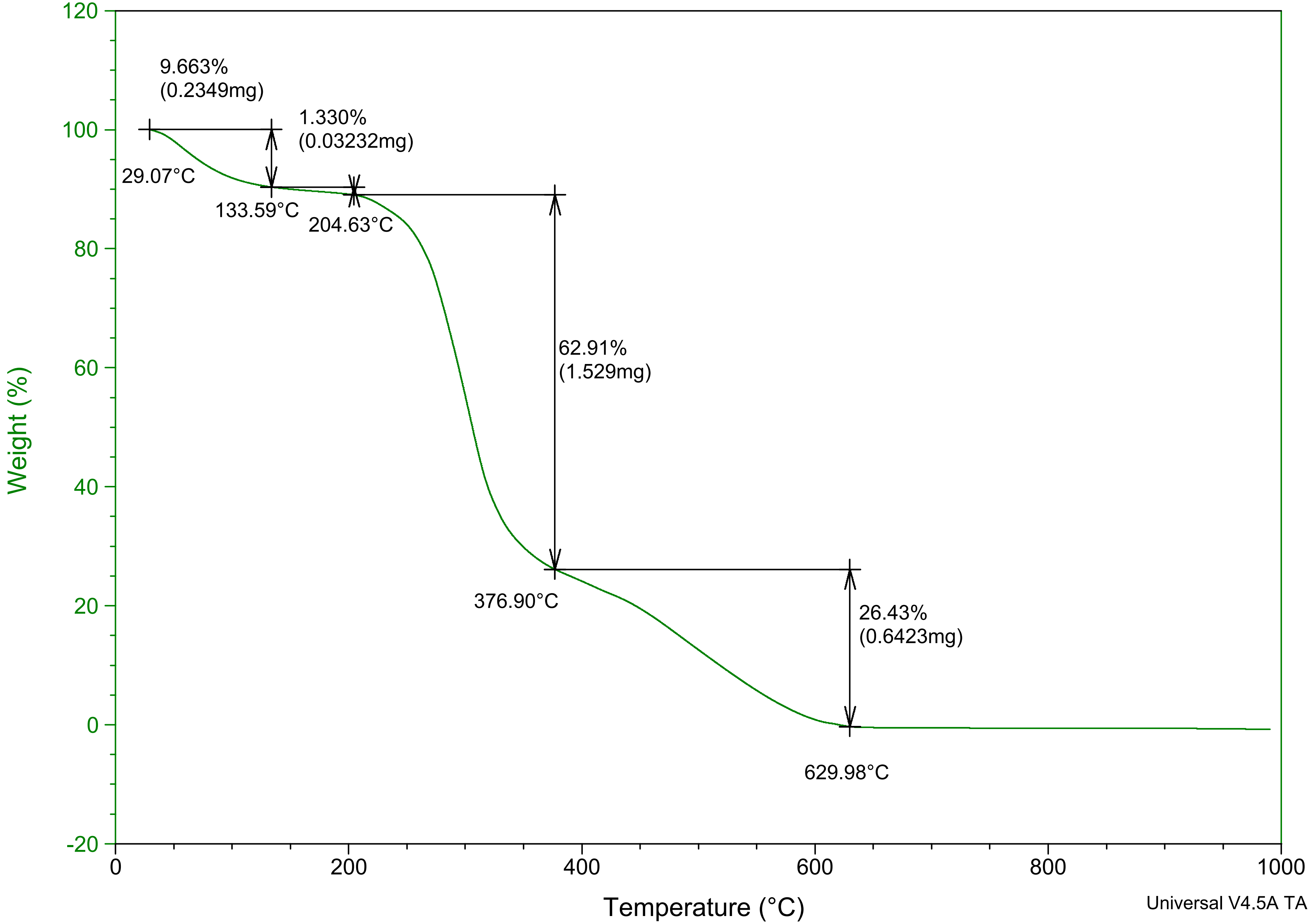

Universal V4.5A TA Instruments

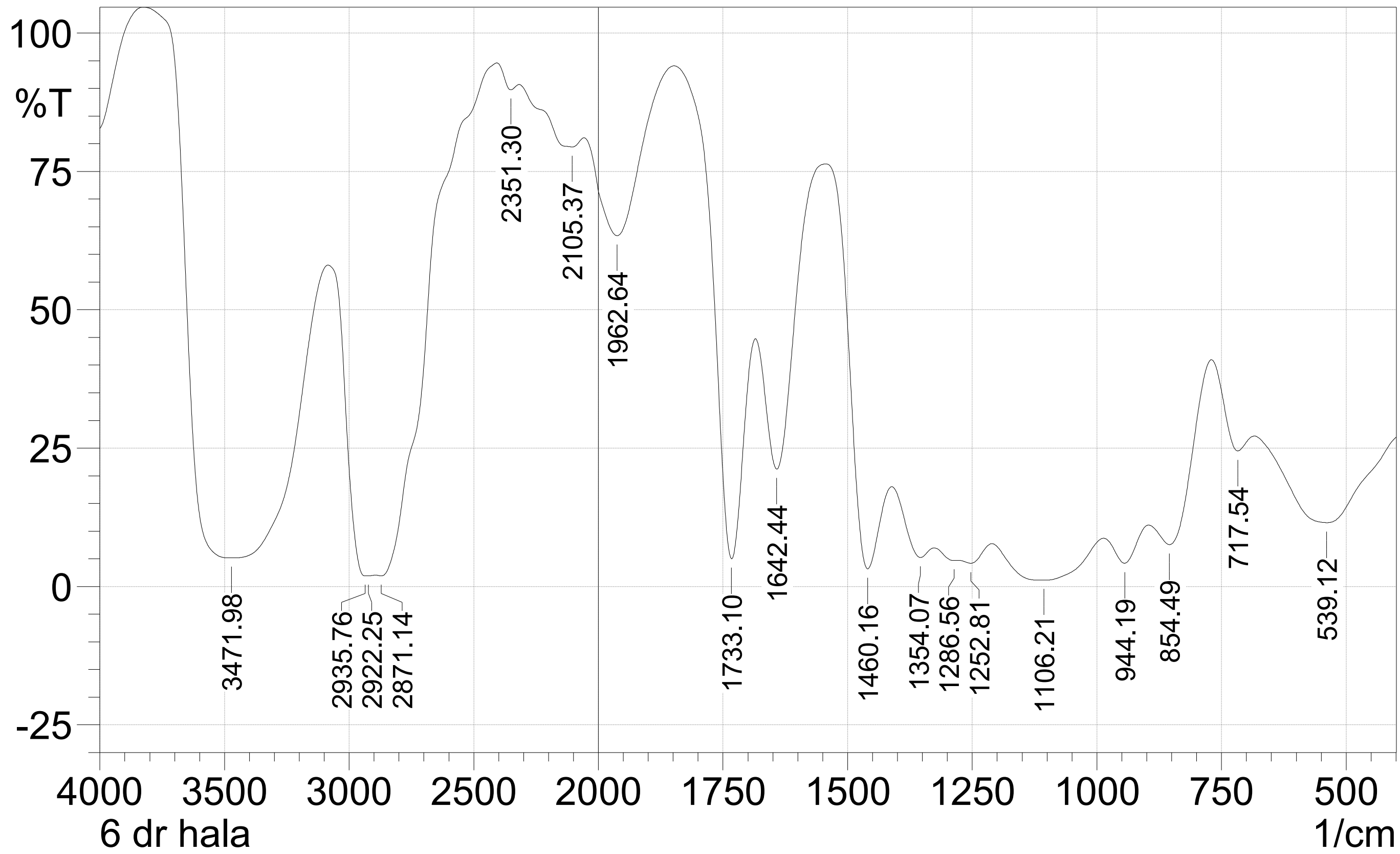

No.	Peak	Intensity	Corr. Intensity	Base (H)	Base (L)	Area	Corr. Area
1	539.12	11.54	15.56	683.79	399.28	214.11	52.8
2	717.54	24.5	8.01	769.62	684.75	45.2	4.71
3	854.49	7.57	13.41	895.96	770.59	102.12	18.84
4	944.19	4.21	5.63	985.66	896.93	103.42	14.11
5	1106.21	1.15	7.05	1210.37	986.62	346.12	103.32
6	1252.81	4.2	1.66	1277.88	1211.34	85.13	4.21
7	1286.56	4.69	0.4	1326.1	1278.85	59.59	0.97
8	1354.07	5.24	5.29	1410.98	1327.07	89.16	9.45
9	1460.16	3.19	36.28	1543.1	1411.94	91.45	36.86
10	1642.44	21.21	33.07	1684.88	1544.07	50.04	18.18
11	1733.1	5.03	54.09	1847.87	1685.84	65.32	37.13
12	1962.64	63.38	23.59	2057.15	1848.83	24.13	11.92
13	2105.37	79.41	3.41	2317.55	2058.11	20.11	2.78
14	2351.3	89.74	2.39	2407.24	2318.51	3.45	0.5
15	2871.14	1.92	4.36	2893.32	2408.21	229.24	3.27
16	2922.25	1.94	0.02	2927.08	2894.28	55.82	0.1
17	2935.76	1.93	2.78	3084.28	2928.04	122.55	2.68
18	3471.98	5.17	77.23	3825.93	3085.24	500.25	421.75

Comment;
6 dr hala

Date/Time; 18/12/2002 11:45:50
No. of Scans;
Resolution;
Apodization;
User; user
